# Analyzing the dynamics of a charged rotating rigid body under constant torques

**DOI:** 10.1038/s41598-024-59857-z

**Published:** 2024-04-29

**Authors:** T. S. Amer, H. F. El-Kafly, A. H. Elneklawy, A. A. Galal

**Affiliations:** 1 Department of Mathematics, Faculty of Science, Tanta University, Tanta, 31527 Egypt; 2 Tanta Higher Institute of Engineering and Technology, Tanta, Egypt; 3 Department of Mathematics, Faculty of Science, Kafrelsheikh University, Kafr El-Sheikh, 33516 Egypt; 4 Engineering Physics and Mathematics Department, Faculty of Engineering, Tanta University, Tanta, 31734 Egypt

**Keywords:** Nonlinear dynamics, Charged rigid body, Numerical methods, Rotational motion, Electromagnetic field, Gyrostatic moment, Applied mathematics, Aerospace engineering

## Abstract

This study explores the dynamical rotary motion of a charged axisymmetric spinning rigid body (RB) under the effect of a gyrostatic moment (GM). The influence of transverse and invariable body fixed torques (IBFTs), and an electromagnetic force field, is also considered. Euler’s equations of motion (EOM) are utilized to derive the regulating system of motion for the problem in a suitable formulation. Due to the lack of torque exerted along the spin axis and the nearly symmetrical nature of the RB, the spin rate is nearly unchanged. Assuming slight angular deviations of the spin axis relative to a fixed direction in space, it is possible to derive approximate analytical solutions (AS) in closed form for the attitude, translational, and rotational movements. These concise solutions that are expressed in complex form are highly effective in analyzing the maneuvers performed by spinning RBs. The study focuses on deriving the AS for various variables including angular velocities, Euler’s angles, angular momentum, transverse displacements, transverse velocities, axial displacement, and axial velocity. The graphical simulation of the subsequently obtained solutions is presented to show their precision. Furthermore, the positive impacts that alterations in the body’s parameters have on the motion’s behavior are presented graphically. The corresponding phase plane curves, highlighting the influence of different values in relation to the electromagnetic force field, the GM, and the IBFTs are drawn to analyze the stability of the body’s motion. This study has a significant role in various scientific and engineering disciplines. Its importance lies in its ability to optimize mechanical systems, explain celestial motion, and enhance spacecraft performance.

## Introduction

In the past century, there has been a significant amount of research focused on addressing Euler’s equations for the RB’s rotational motion. This involves studying the forces and torques acting on the body, applying rotational dynamics principles, and accurately calculating relevant quantities and parameters to describe the body’s rotation. Numerous researchers have been attentive to this topic, as shown in Refs.^1–21^. The governing EOM for such a problem has been approached in all of the aforementioned references especially in Refs.^1,2^. The formulations for the equations of Euler’s angles with their different sequences have been presented in Ref.^3^. In Ref.^4^, the author obtained an estimated analytic solution for the rotary motion of a nearly symmetrical RB that experiences IBFTs. The approximate solution for Euler’s EOM is precisely expressed using Fresnel integrals^5^ and is accurate for bodies with symmetry. A rough resolution for the Euler’s angles is determined with Fresnel integrals, as well as sine and cosine integrals. In Ref.^6^, the author utilized a methodology to determine the necessary solution for Euler’s EOM, where the angular velocity components of RB solely rely on the time parameter. The essential solutions were provided through analytical formulas using both time-dependent and real-valued coefficients. The confirmed coefficients are the solutions to a mutual system consisting of two Riccati ordinary differential equations. The rotary motion of the RB with a Lagrangian mass distribution is investigated in Ref.^7^. The researcher assumes that the RB has a high angular velocity and that the perturbing moments are much smaller in comparison to the gravitational moment. The study utilized the acceleration method with the introduction of a small parameter in a unique manner. In Ref.^8^, the authors examined the rotary motion behavior of the RB according to Lagrange’s case when it is subjected to slowly varying, restoring, and perturbing torques or forces. The problem is solved using typical solution steps. By applying the asymptotic technique, the researchers obtained the averaged EOM, resulting in some qualitative results.

In Ref.^9^, the motion of the RB is analyzed when subjected to potential force and a GM. It was found that the EOM has three extra solutions. It was observed that if the body mass distribution in a heavy gyrostat case fulfills Kovalevskaya and Goryachev–Chaplygin’s generalized conditions, a solution to the problem can be obtained. In Ref.^10^, the authors delved into exploring the potential of employing an electrodynamic control system for maintaining the monaxial orientation of an artificial satellite in the orbital coordinate frame. A theorem on the asymptotic stability of body-controlled attitude motion is presented, and its effectiveness through numerical modeling with distributed delay is demonstrated. In a recent study^11^, the movement of a body point in the proximity of an attractive center is explored. The authors effectively demonstrated the analogy between the trajectory of the body point in space and the orbital of an electron in a hydrogen atom. It is important to note that all the theoretical discoveries made in this research are novel, as for the first time, researchers are investigating the movement of a body point close to a center of attraction. In Ref.^12^, the use of a large parameter is implemented to demonstrate the asymptotic solutions of Euler’s EOM for a restricted gyrostatic system. Additionally, the derived solutions are supported by simulated diagrams, showcasing their accuracy. The Barnett-London effect and the motion of the RB were considered in Ref.^13^. To address the problem of a gyrostat moving in a central Newtonian force field (NFF), the author developed a generalized method. Surprisingly, the generalized solution for the RB moving by inertia in a perfect incompressible fluid is a new specific solution. The Hamiltonian function is employed to express the EOM within the Lie–Poisson system^14^. Moreover, the stability criteria were stated using the energy-casimir technique^15^**,** and the author utilized the linear approximation method to analyze the conditions for instability for these equilibria. In Ref.^16^, specific applications of RB’s motion in space are explored. It examined how the body rotates in the presence of the NFF, gravitational force, and GM. Additionally, the stability of a single-rotor gyrostat rotating without any external constraints has been investigated. In Ref.^17^, the author discovered that the GM’s presence could have a notable impact on the control of the RB’s rotational motion. To acquire the necessary analytical expressions for variable control torques, the author employed Poincaré’s small parameter method to obtain solutions for the EOM. Another investigation of the RB’s problem was found in Ref.^18^ according to the influence of the GM and NFF, where the first component of the GM was given a zero value. In order to handle situations involving irrational frequencies, Euler’s EOM was solved using Poincaré’s small parameter method, leading to asymptotic solutions. An analysis of the influence of GM, IBFTs, and resistant forces on the motion of a charged RB is given in Ref.^19^. A suitable governing system for EOM is approached using the averaging method. To reach the required results, Taylor’s method is used along with some initial conditions to solve the averaged EOM of the RB. Various values related to GM, electromagnetic force field, IBFTs, and torque of a resistive force are illustrated with diagrams. It has also been discussed whether the motion of the RB is stable or not. In Ref.^20^, the analysis focuses on the equilibrium attitude and stability of a rigid spacecraft orbiting a uniformly rotating asteroid while on a stationary orbit. The linearized EOMs are derived under the assumption of small motions. The equilibrium attitude is then determined for both a general spacecraft and a symmetrical spacecraft. The equilibrium attitude is found to deviate slightly from zero Euler angles due to the higher-order inertia integrals of the spacecraft. The stability of this conservative system is examined by analyzing the necessary conditions for stability using the linearized equations of motion. In Ref.^21^, the concept of attitude stability is expanded to include a rigid spacecraft orbiting a uniformly rotating asteroid while remaining stationary. This extended problem is examined using the linearized EOMs, taking into account the harmonic coefficients of the asteroid’s gravity field. In Ref.^22^, the authors discovered that the nonlinear stability domain of a spacecraft positioned on the intermediate axis of an asteroid can vary significantly from the traditional Lagrange region found in a circular orbit within a central gravity field. By utilizing the Lie group framework established by geometric mechanics, along with techniques like the energy-Casimir method for nonlinear stability and variational integrators for enhanced numerical simulation accuracy, researchers can gain valuable insights into equilibrium determination and control problems through the lens of geometric control theory. In Ref.^23^, the authors’ goal was to investigate the rotational movement of an asymmetric RB subjected to IBFTs and a non-zero first component GM. They employed Euler’s EOMs to derive a series of dimensionless equations, which were then suggested for analyzing the stability of equilibrium points. In Ref.^24^, analytic solutions are derived for a near-symmetric RB subject to time-varying torques. The resulting solutions of Euler’s EOM are considered to be exact. A systematic approach was demonstrated for assessing these integrals in a concise and precise form. In Ref.^25^, solutions to the RB’s problem were obtained through an analytical method for the scenario where a rotating RB experiences an axial time-varying torque. These solutions provide a description of the overall attitude movement of a close-symmetric RB when subjected to time-varying torques around each of its three body-fixed axes. In Ref.^26^, the study focuses on the shortest duration of the 3D movement of an unbalanced RB, which is subject to a rotational force that is caused by viscous friction and gyrostatic forces. The initial position of the center of mass was at the Cartesian frame’s original point, where an optimal control method is formulated for this scenario. The movement of a gyrostat that possesses a symmetrical charge is investigated in Ref.^27^ when the gyrostat’s center of mass is slightly displaced from the axis of dynamic symmetry. A uniform electromagnetic force field and a GM are acting on the gyrostat’s motion. The body’s EOM is derived and solved when the restriction of irrational frequencies is considered.

The examination of the impact of GM on the rotary motion of a spinning axisymmetric charged RB is provided in this study. Additionally, the influence of transverse and IBFTs and electromagnetic force field are considered. Euler’s equation is used to derive the EOM that governs the RB’s rotation. Due to the lack of torque exerted along the spin axis and the RB possesses a nearly symmetrical structure, the spin rate remains almost constant. Assuming small angular deviations of the spin axis regarding a stationary direction in space, it is possible to obtain approximate AS in closed form for the attitude, translational, and rotational movements. These concise solutions, expressed in complex form, are highly effective in analyzing the maneuvers performed by spinning RBs. The approach focuses on deriving the AS for various variables including angular velocities, Euler’s angles, angular momentum, transverse velocities, transverse displacements, axial velocity, and axial displacement. To demonstrate the accuracy of the method used, graphical simulations of these solutions are presented. Furthermore, a computer program is used to diagram these results, showing the positive influence of various values for the body’s parameters on the motion’s behavior. The diagrams include phase plane level curves and show how the GM, charge, and IBFTs affect the motion. Therefore, the stability of the RB’s motion through the analysis of phase plane diagrams has been discussed. The study plays a crucial role in multiple scientific and engineering fields, as it enables the optimization of mechanical systems, provides explanations for celestial motion, and enhances the performance of spacecraft.

## The problem’s articulating

**Figure 1:**
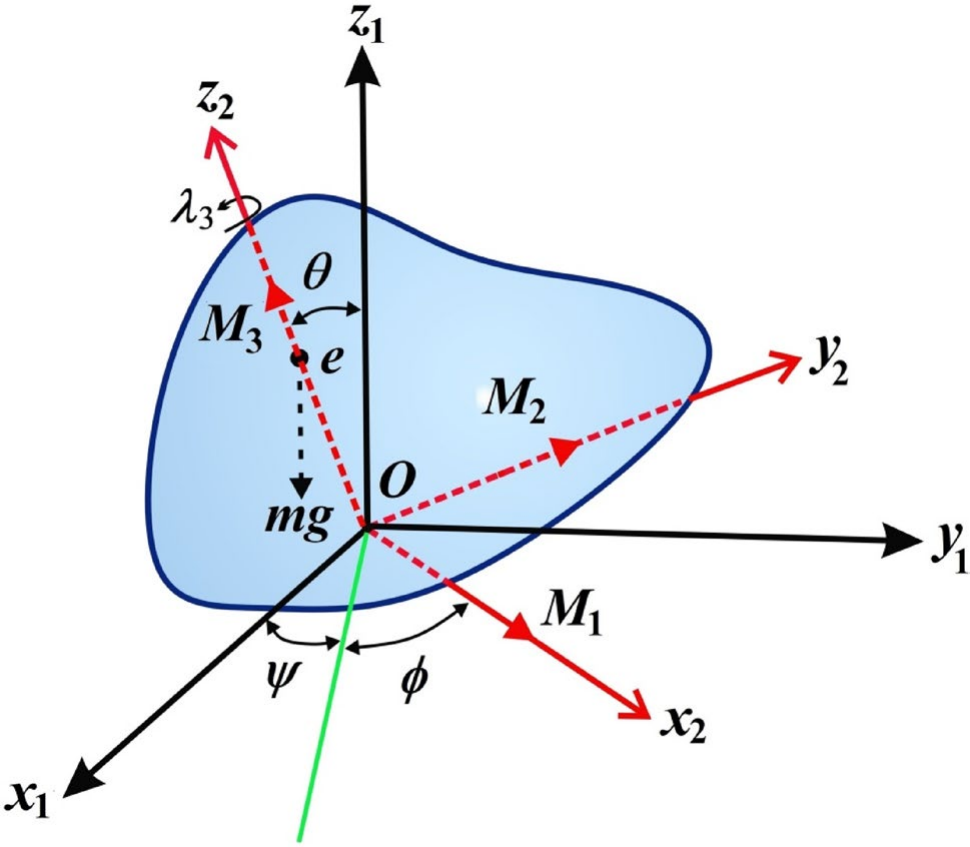
The structure of the RB’s model.

This section focuses on analyzing the rotary motion of a spinning charged RB about its inertia center. Therefore, two Cartesian frames are considered: the first frame, $Ox_{1} y_{1} z_{1}$, out remains fixed, while the second frame, $Ox_{2} y_{2} z_{2}$, is rotating and aligned with the system’s inertia center. This rotation corresponds to the rotation of the body itself, as shown in Fig. 1. Assume that the vector $\underline{\Omega } = (p,q,r)$ represents the angular velocity of the body, with its components aligned with the body’s principal axes. Let the vector $\underline {D} = (D_{1} ,D_{2} ,D_{3} )$ denote the inertia tensor of the body whose components are directed on the same axes. It is important to consider that the body is influenced by a vector $\underline{\lambda } = (\lambda_{1} ,\lambda_{2} ,\lambda_{3} )$, directed along these axes, wherein its third component, $\lambda_{3}$, carries a non-zero value, while the remaining components, $\lambda_{1}$ and $\lambda_{2}$, maybe zero, and the electromagnetic force field of a strength $H$ which is due to the charged body as a result of a point charge $e$ at a distance $l$ from the origin $O$ which makes an angle $\sigma$ with the fixed axis $Oz_{1}$.

Therefore, Euler’s EOM governing the RB’s motion is given by^1,2^

1 $$\begin{aligned} D_{1} \dot{p} & + (\,D_{3} - D_{2} \, )qr + (\lambda_{3} \, - eHl^{2} \cos \sigma )q = M_{1} , \\ D_{2} \dot{q} & + (D_{1} \, - D_{3} \,)\, \, rp - (\lambda_{3} - eHl^{2} \cos \sigma )p\, = M_{2} , \\ D_{3} \, \dot{r} & + (\,D_{2} - D_{1} \,)\, \, pq = M_{3} , \\ \end{aligned}$$

where $M_{j} \,(j = 1,2,3)$ are the IBFTs, and the dot represents the differentiation concerning time $t$.

## The analytical solutions to some parameters controlling the problem

This section presents a detailed AS for some parameters that control the motion of the body as follows.

### The angular velocities solutions

One can observe that system (1) may be rewritten in the form

2 $$\begin{aligned} \dot{p} &= D_{1}^{ - 1} \Bigg\{ M_{1} - q\Bigg[(D_{3} - D_{2} )r + (\lambda_{3} \, - eHl^{2} \cos \sigma )\Bigg]\Bigg\} , \hfill \\ \dot{q} &= D_{2}^{ - 1} \Bigg\{ M_{2} - p\Bigg[(D_{1} - D_{3} \, )r - (\lambda_{3} \, - eHl^{2} \cos \sigma )\Bigg]\Bigg\} , \hfill \\ \, \dot{r} &= D_{3}^{ - 1} \Bigg[M_{3} - (\,D_{2} - D_{1} \, )pq\Bigg]. \hfill \\ \end{aligned}$$

Considering the assumption that the RB is rotating around its $z_{2}$-axis, it can be inferred that $M_{3}$ will equal zero. In the case of an axisymmetric RB, where $D_{1}$ is equal to $D_{2}$, or in a situation where the difference between $D_{1}$ and $D_{2}$ is negligible, the third equation of system (2) can be expressed as

3 $$r \approx r_{0} = r(0).$$

Now, introducing new variables such as

4 $$\begin{aligned} &\zeta_{1} = {{(D_{3} - D_{2} )} \mathord{\left/ {\vphantom {{(D_{3} - D_{2} )} {D_{1} }}} \right. \kern-0pt} {D_{1} }},\,\,\,\,\,\,\,\zeta_{2} = {{(D_{3} - D_{1} )} \mathord{\left/ {\vphantom {{(D_{3} - D_{1} )} {D_{2} }}} \right. \kern-0pt} {D_{2} }},\,\,\,\,\,\,\,\,\zeta = \sqrt {\zeta_{1} \zeta_{2} } , \hfill \\ &{p = p_{1} \mathord{\left/ {\vphantom {{p = p_{1} } {\sqrt {\,\zeta_{2} } }}} \right. \kern-0pt} {\sqrt {\,\zeta_{2} } }},\,\,\,\,\,\,\,\,{{q = q_{1} } \mathord{\left/ {\vphantom {{q = q_{1} } {\sqrt {\,\zeta_{1} } }}} \right. \kern-0pt} {\sqrt {\,\zeta_{1} } }}. \hfill \\ \end{aligned}$$

Then, the first two equations in the system (2) may be written as follows

5 $$\begin{aligned} \dot{p}_{1} = & D_{1}^{ - 1} \Bigg\{ {M_{1} \sqrt {\zeta_{2} } - q_{1} \Bigg[D_{1} \zeta r_{0} + (\lambda_{3} \, - eHl^{2} \cos \sigma ){{(\sqrt {\zeta_{2} } } \mathord{\left/ {\vphantom {{(\sqrt {\zeta_{2} } } {\sqrt {\zeta_{1} } }}} \right. \kern-0pt} {\sqrt {\zeta_{1} } }})\Bigg]} \Bigg\}, \\ \dot{q}_{1} = & D_{2}^{ - 1} \Bigg\{ {M_{2} \sqrt {\zeta_{1} } + p_{1} \Bigg[D_{2} \zeta r_{0} + (\lambda_{3} \, - eHl^{2} \cos \sigma ){{(\sqrt {\zeta_{1} } } \mathord{\left/ {\vphantom {{(\sqrt {\zeta_{1} } } {\sqrt {\zeta_{2} } }}} \right. \kern-0pt} {\sqrt {\zeta_{2} } }})\Bigg]} \Bigg\}. \\ \end{aligned}$$

Defining the following new variables once more

6 $$A_{1} = {{M_{1} \sqrt {\,\zeta_{2} } } \mathord{\left/ {\vphantom {{M_{1} \sqrt {\,\zeta_{2} } } {D_{1} }}} \right. \kern-0pt} {D_{1} }},\,\,\,\,A_{2} = {{M_{2} \sqrt {\,\zeta_{1} } } \mathord{\left/ {\vphantom {{M_{2} \sqrt {\,\zeta_{1} } } {D_{2} }}} \right. \kern-0pt} {D_{2} }}.$$

As a result, system (5) turns into

7 $$\begin{aligned} \dot{p}_{1} = & A_{1} - q_{1} \Bigg\{ \zeta r_{0} + {{\Bigg[\zeta (\lambda_{3} \, - eHl^{2} \cos \sigma )} \mathord{\left/ {\vphantom {{\Bigg[\zeta (\lambda_{3} \, - eHl^{2} \cos \sigma )} {\zeta_{1} D_{1} }}} \right. \kern-0pt} {\zeta_{1} D_{1} }}\Bigg]\Bigg\} , \\ \dot{q}_{1} = & A_{2} + p_{1} \Bigg\{ \zeta r_{0} + \Bigg[{{\zeta (\lambda_{3} \, - eHl^{2} \cos \sigma )} \mathord{\left/ {\vphantom {{\zeta (\lambda_{3} \, - eHl^{2} \cos \sigma )} {\zeta_{2} D_{2} }}} \right. \kern-0pt} {\zeta_{2} D_{2} }}\Bigg]\Bigg\} . \\ \end{aligned}$$

At $D_{1} \approx D_{2}$ in a nearly axisymmetric RB, multiplying the second equation of (7) by $i = \sqrt { - 1}$ and adding it to the first equation, one can obtain

8 $$\dot{p}_{1} + i\dot{q}_{1} = A_{1} + iA_{2} + i\zeta \Bigg[ {r_{0} + \left\{ {\left( {\lambda _{3} {\kern 1pt} - eHl^{2} \cos \sigma } \right)\Bigg/\zeta _{1} D} \right\}_{1} } \Bigg](p_{1} + iq_{1} ),$$

which can be rewritten as

9 $$\dot{\Pi }_{1} - i\zeta \Bigg[ {r_{0} + \left\{ {\left( {\lambda _{3} {\kern 1pt} - eHl^{2} \cos \sigma } \right)\Bigg/\zeta _{1} D_{1} } \right\}} \Bigg]\Pi _{1} = A.$$

Here,

10 $$A = A_{1} + iA_{2} ,\,\,\Pi_{1} = p_{1} + iq_{1} .$$

The novel solution of Eq. (9) is presented as follows

11 $$\begin{aligned} \Pi_{1} (t) = & \Pi_{10} e^{{i\zeta t\Bigg[r_{0} \, + \Bigg\{ \,{{(\lambda_{3} \, - eHl^{2} \cos \sigma )} \mathord{\left/ {\vphantom {{(\lambda_{3} \, - eHl^{2} \cos \sigma )} {\zeta_{1} D_{1} \Bigg\} }}} \right. \kern-0pt} {\zeta_{1} D_{1} \Bigg\} }}\Bigg]}} + \frac{{iA}}{{\zeta\Bigg[r_{0} \, + \Bigg\{ \,{{(\lambda_{3} \, - eHl^{2} \cos \sigma )} \mathord{\left/ {\vphantom {{(\lambda_{3} \, - eHl^{2} \cos \sigma )} {\zeta_{1} D_{1} \Bigg\} }}} \right. \kern-0pt} {\zeta_{1} D_{1} \Bigg\} }}\Bigg]}} \\ & \times \Bigg[1 - e^{{i\zeta t\Bigg[r_{0} \, + \Bigg\{ \,{{(\lambda_{3} \, - eHl^{2} \cos \sigma )} \mathord{\left/ {\vphantom {{(\lambda_{3} \, - eHl^{2} \cos \sigma )} {\zeta_{1} D_{1} \Bigg\} }}} \right. \kern-0pt} {\zeta_{1} D_{1} \Bigg\} }}\Bigg]}} \Bigg], \\ \end{aligned}$$

where $\Pi_{10} = \Pi_{1} (0)$, the right-hand side of Eq. (11) contains the homogeneous and nonhomogeneous solutions of Eq. (10). Comparing the real and imaginary terms on both sides of Eq. (11) gives

12 $$p_{1} = {\text{Re}} \Bigg[\Pi_{1} (t)\Bigg],\,\,\,\,\,\,\,\,\,\,\,q_{1} = {\text{Im}} \Bigg[\Pi_{1} (t)\Bigg].$$

The symbols ${\text{Re}}$ and ${\text{Im}}$ are used to represent the real and imaginary components, respectively. Therefore, the transversal angular velocities have the forms

13 $$\begin{gathered} p = {{{\text{Re}} \Bigg[\Pi_{1} (t)\Bigg]} \mathord{\left/ {\vphantom {{{\text{Re}} \Bigg[\Pi_{1} (t)\Bigg]} {\sqrt {\zeta_{2} } }}} \right. \kern-0pt} {\sqrt {\zeta_{2} } }} = {{\Bigg[\Pi_{1} (t) + \tilde{\Pi }_{1} (t)\Bigg]} \mathord{\left/ {\vphantom {{\Bigg[\Pi_{1} (t) + \tilde{\Pi }_{1} (t)\Bigg]} {2\sqrt {\zeta_{2} } }}} \right. \kern-0pt} {2\sqrt {\zeta_{2} } }}, \hfill \\ q = {{{\text{Im}} \Bigg[\Pi_{1} (t)\Bigg]} \mathord{\left/ {\vphantom {{{\text{Im}} \Bigg[\Pi_{1} (t)\Bigg]} {\sqrt {\zeta_{1} } }}} \right. \kern-0pt} {\sqrt {\zeta_{1} } }} = {{\Bigg[\Pi_{1} (t) - \tilde{\Pi }_{1} (t)\Bigg]} \mathord{\left/ {\vphantom {{\Bigg[\Pi_{1} (t) - \tilde{\Pi }_{1} (t)\Bigg]} {2\sqrt {\zeta_{1} } }}} \right. \kern-0pt} {2\sqrt {\zeta_{1} } }}, \hfill \\ \end{gathered}$$

whereas $\tilde{\Pi }_{1} (t)$ is the complex conjugate of the function $\Pi_{1} (t)$.

So, take into account the subsequent information for the values of parameters during all upcoming sections as follows

$$\begin{array}{*{20}l} D( = 8.7,8.6,11.1)\,{\text{kg}}\,{\text{m}}^{2} ,\,\,\,\,m = 10\,{\text{kg}},\,\,\,e( = 0.001,0.003,0.005){\text{C}},\,\,\,\,M_{1} ( = 4.0,4.5,5.0)\,{\text{N}}\,{\text{m}}, \hfill \\ M_{2} = M_{3} = 0,\,\,\,\,f_{1} = f_{2} = 0,\,\,\,\,f_{3} = 3{\text{N}},\,\,\,\,\lambda_{3} ( = 1.3,1.6,1.9)\,{\text{kg}}\,{\text{m}}^{2} \,{\text{s}}^{ - 1} ,\,\,\,\,H = 100\,{\text{T}}, \hfill \\ l = 1.02\,{\text{m}},\,\,\,\,\theta = 0.37\,{\text{rad}}, \hfill \\ \end{array}$$

while the RB’s motion is initiated with

$$\begin{array}{*{20}l} \omega_{1} (0) = \omega_{2} (0) = \omega_{3} (0) = 0,\,\,\,\,\,p(0) = q(0) = 0,\,\,\,\,\,\psi (0) = \phi (0) = \theta (0) = 0, \hfill \\ r(0) = 2.7\;{\text{rpm,}}\,\,\,\,\eta_{1} (0) = \eta_{2} (0) = \eta_{3} (0) = 0, \hfill \\ \end{array}$$

and it is investigated over the whole-time interval $$t \in \Bigg[0,50\Bigg]$$$$\;{\text{s}}$$.

**Figure 2:**
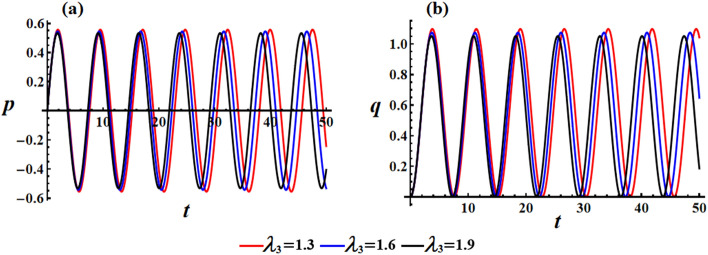
Presents the diagrams of AS at $e = 0.003\,{\text{C,}}\,\,M_{1} = 4.0\,{\text{N}}\,{\text{m}}$ when $\lambda_{3} ( = 1.3,1.6,1.9)\,{\text{kg}}\,{\text{m}}^{2} \,{\text{s}}^{ - 1}$: (**a**) the angular velocity $p$, (**b**) the angular velocity $q$.

**Figure 3:**
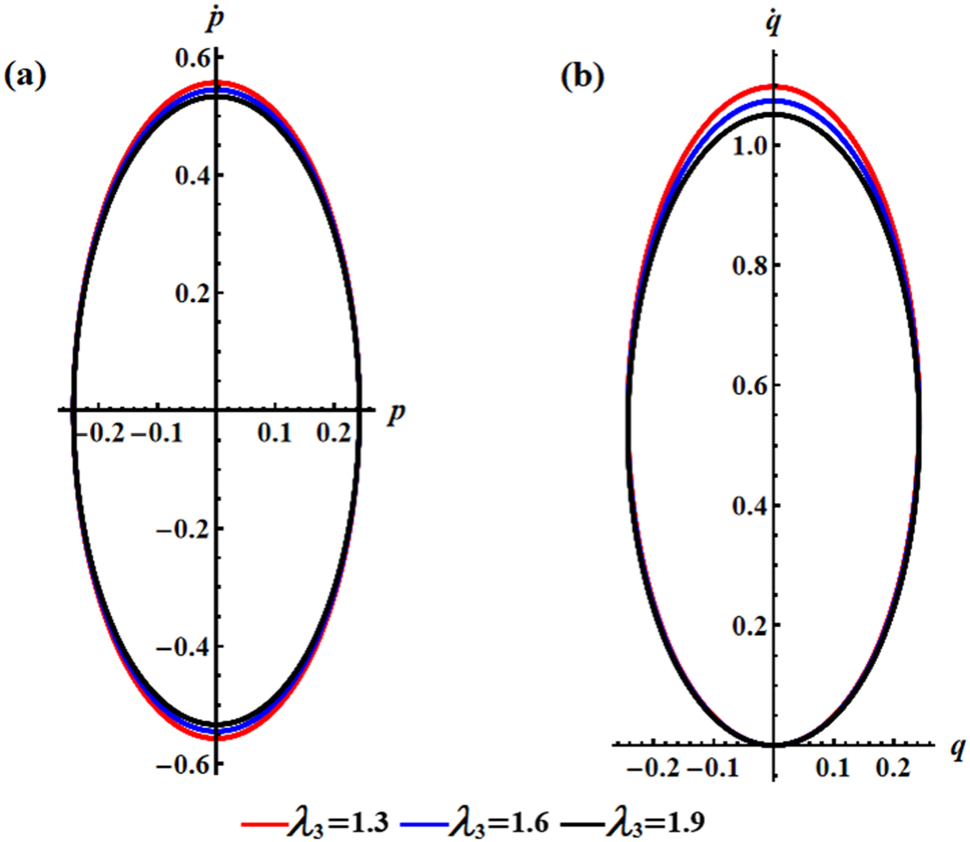
Shows the curves of the phase plane for the solutions in Fig. 2: (**a**) in the plane $p\dot{p}$ and (**b**) in the plane $q\dot{q}$.

We studied the positive impact of GM’s third component on the solutions on the behavior of the studied model when $\lambda_{3}$ varies between three distinct values, i.e., $\lambda_{3} ( = 1.3,1.6,1.9){\text{ kg m}}^{2} {\text{ s}}^{ - 1}$ at $e = 0.003\,{\text{C,}}\,\,M_{1} = 4.0\;{\text{N}}\;{\text{m}}$. In Fig. 2, the AS for angular velocities $p$ and $q$ mentioned in Eq. (13) are displayed. The graphs demonstrate the impact of the GM on the obtained solutions. It is apparent that an increase in GM’s third component results in a decrease in the amplitude and wavelength of the plotted waves, while the fluctuations number increases slightly, as seen in Fig. 2a,b. This ultimately results in a decrease in the values of the angular velocities over the examined time interval. The related phase plane plots of these solutions are explored in parts of Fig. 3. The included curves have forms of symmetric closed ellipsoid trajectories, in which the graphed trajectories remain stable as $\lambda_{3}$ varies. In part (a) of the figure, it is shown that increasing the GM reduces the trajectory in both positive and negative regions. However, in part (b), the trajectory is contained within the positive region only. The reason is due to the solution’s mathematical form (13).

**Figure 4:**
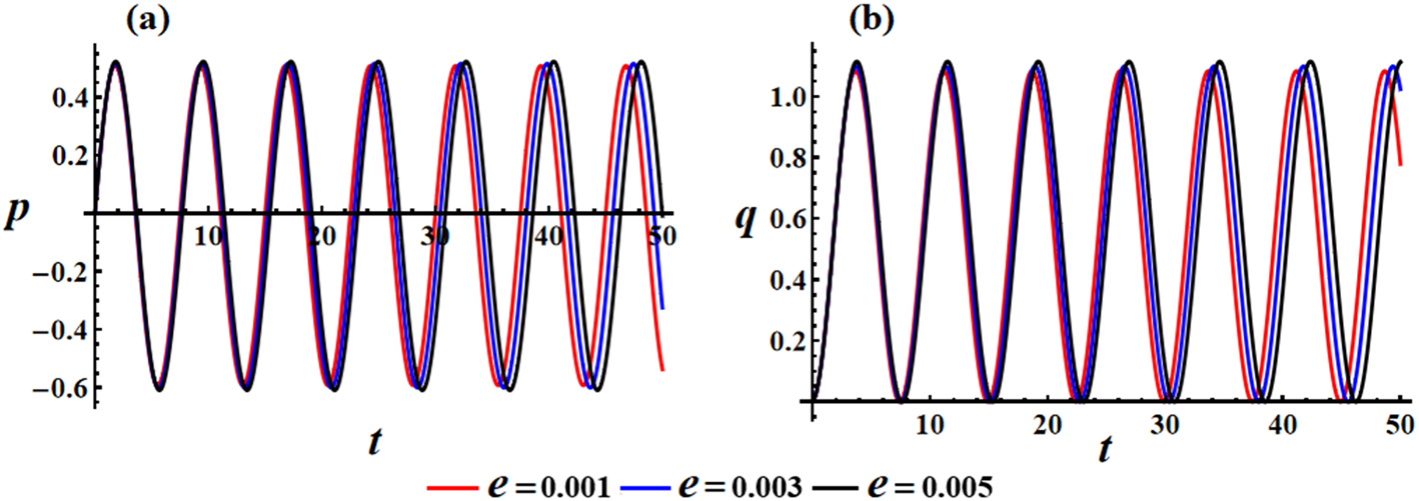
Sketches the influence of $e( = 0.001,0.003,0.005){\text{C}}$ on the behavior of (**a**) the solution $p$, (**b**) the solution $q$ at $\lambda_{3} = 1.3\;{\text{kg}}\;{\text{m}}^{2} \;{\text{s}}^{ - 1} ,\,M_{1} = 4.0\;{\text{N}}\;{\text{m}}$.

**Figure 5:**
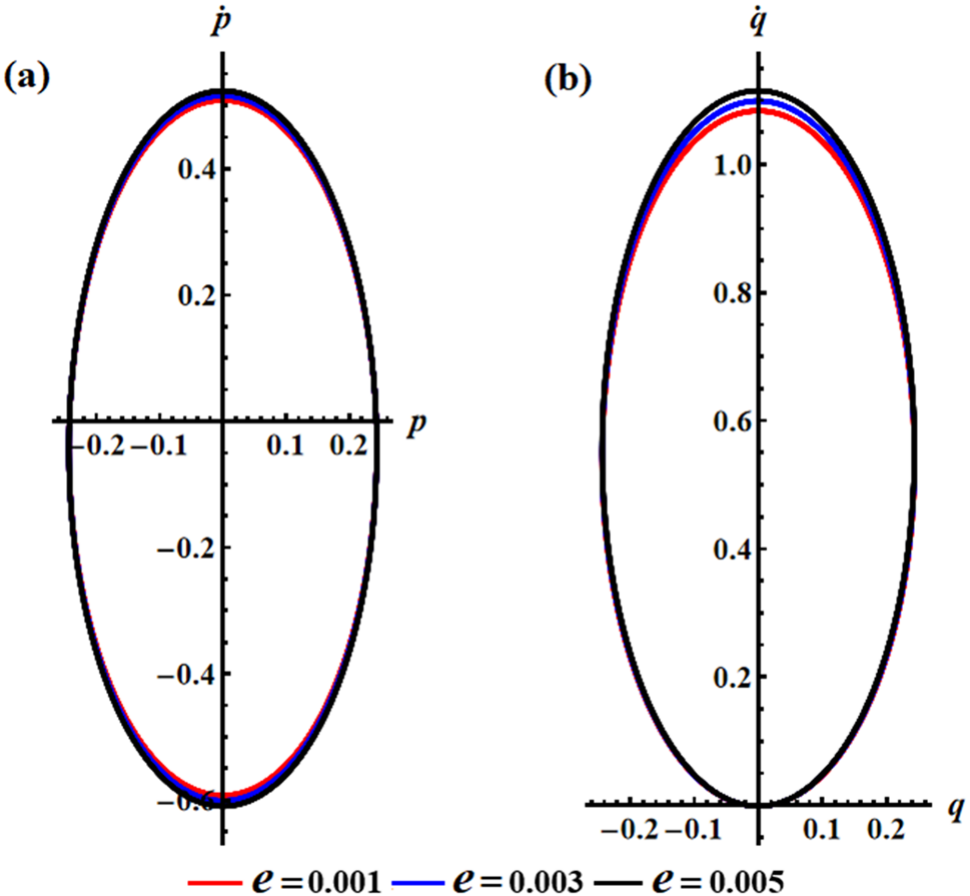
Shows the phase plane’s curves for the same considered values in Fig. 4: (**a**) in the plane $p\dot{p}$ and (**b**) in the plane $q\dot{q}$.

The effect of the electromagnetic force field is due to the presence of a point charge on the $z_{1}$ axis, then its action on the motion’s behavior is investigated according to the varied value of $e$ i.e., $e( = 0.001,0.003,0.005){\text{C}}$ at $\lambda_{3} = 1.3\,{\text{kg}}\,{\text{m}}^{2} \,{\text{s}}^{ - 1} {,}\,\,M_{1} = 4.0\,{\text{N}}\,{\text{m}}$. Therefore, Fig. 4 is graphed to illustrate the temporal behavior of the angular velocities’ solutions $p$ and $q$ when $e$ has the aforementioned values. As the charge values $e$ increase, the amplitude and wavelength of the plotted waves increase while their wavelengths decrease, as indicated in parts (a) and (b) of Fig. 4. This ultimately affects the RB’s behavior by causing an increase or decrease in its angular velocities according to $e$ values. Moreover, these presented waves in Fig. 4a have a symmetrical form about the horizontal time axis, i.e., they oscillate between the positive and negative values of the solutions $p$, while the presented waves in Fig. 4b have positive values during the examined time interval. The reason backs to the mathematical forms of the expressions $p$ and $q$. The phase plane diagrams depicting these solutions are graphed in Fig. 5, in which they illustrate typical closed ellipsoid trajectories. These trajectories remain stable regardless of the variation in $e$. In Fig. 5a, the trajectories expand in both positive and negative regions. However, in Fig. 5b, the trajectories become confined to be in the positive region only.

**Figure 6:**
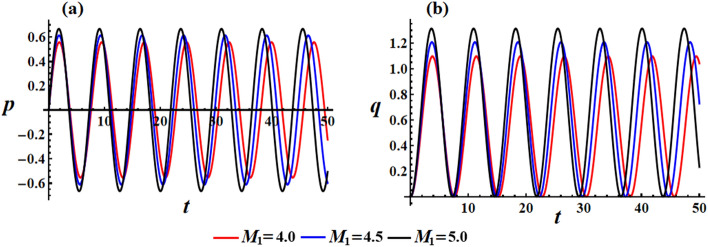
Highlights the beneficial influence of $M_{1}$ values on the behavior of (**a**) the solution $p$, (**b**) the solution $q$.

**Figure 7:**
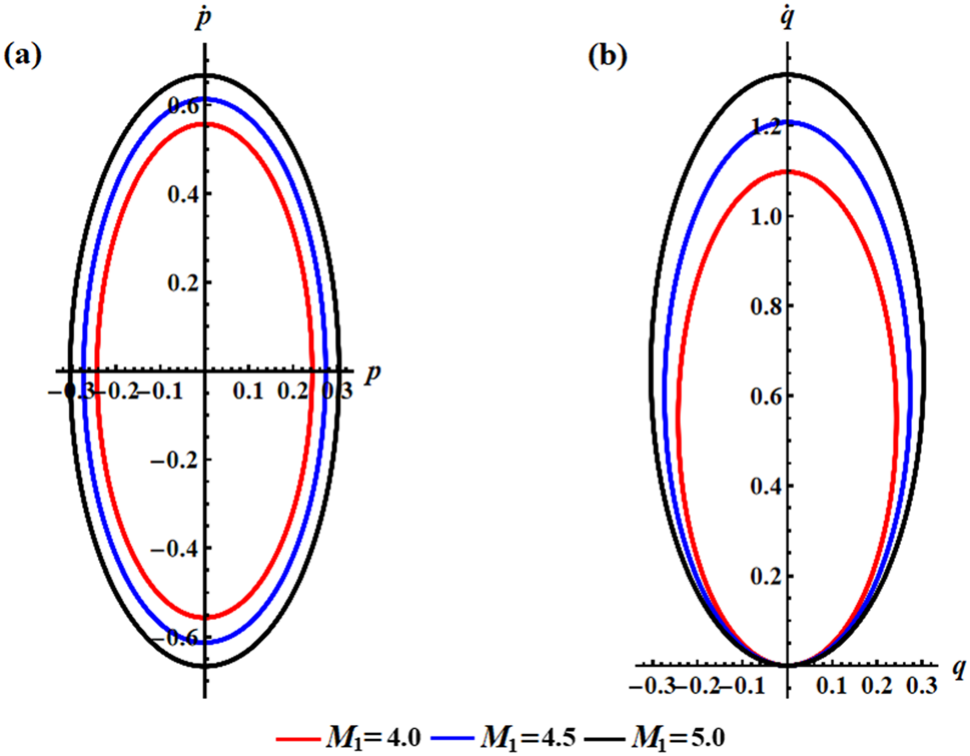
Presents the curves of the phase plane for the identical values depicted in Fig. 6: (**a**) in the plane $p\dot{p}$ and (**b**) in the plane $q\dot{q}$.

We can investigate on how the solutions for the RB’s problem are influenced by the first component of the IBFT, as $M_{1}$ varies according to the distinct values $M_{1} ( = 4.0,4.5,5.0)\,{\text{N}}\,{\text{m}}$ at $\lambda_{3} = 1.3\,{\text{kg}}\,{\text{m}}^{2} \,{\text{s}}^{ - 1} {,}\,\,e = 0.003{\text{C}}$. The impact of these values on the angular velocities $p$ and $q$ can be seen clearly in Fig. 6. The graphs show that as the value of $M_{1}$ increases, the amplitude and wavelength of the solution waves decrease while the frequency increases, and then the oscillations number increases. Looking at the depicted curves in this illustration, it can be inferred that the provided solutions are expected to be stable and devoid of chaos. In order to validate this anticipation, the phase plane diagrams depicting the relationship between the solutions and their respective first derivatives are illustrated in Fig. 7. Symmetrical closed curves have been observed in both parts (a) and (b) of this illustration. These curves demonstrate the consistent behavior of the obtained solutions throughout the analyzed interval, which can be attributed to their periodic nature. The depicted curves in Fig. 7a illustrate that as the IBFT is increased, there is a decrease in the area between the observed trajectories in both the positive and negative regions. Additionally, Fig. 7b shows a reduction, specifically in the trajectory within the positive region.

### Eulerian angles solutions

The 3-1-2 Euler angle sequence^3^, consisting of Euler’s angles $\psi ,\phi ,$ and $\theta$, is used to describe the process of elucidating the correspondence between the reference frame attached to the body and the reference frame that remains fixed in space, as seen in Fig. 1. The associated kinematic equations are as follows

14 $$\begin{aligned} \dot{\psi } = & p\cos \phi + r\sin \phi , \\ \dot{\phi } = & q - (r\cos \phi - p\sin \phi )\tan \psi , \\ \, \dot{\theta } = & (r\cos \phi - p\sin \phi )\sec \psi . \\ \end{aligned}$$

The equations at hand are extremely complex and appear to be difficult to solve. However, significant advancements have been achieved by employing linearization techniques, such as assuming that the variables $\psi$ and $\phi$ are small.

Additionally, small-angle approximations can be used for $\psi$ and $\phi$, and it is further assumed that $\phi p$ is considerably smaller than $r$. As a result of these simplifications, system (14) is simplified to

15 $$\begin{aligned} \dot{\psi } \approx & p + r\phi , \\ \dot{\phi } \approx & q - r\psi , \\ \, \dot{\theta } \approx & r. \\ \end{aligned}$$

The solution of the third equation in (15) is

16 $$\theta (t) = r_{0} \,t + \theta_{0} ,$$

where $\theta_{0} = \theta (0)$. Multiplying the second equation in (15) by $i$ and adding the results to the first equation, yields

17 $$\dot{\psi } + i\dot{\phi } = p + iq - ir_{0} (\psi + i\phi ).$$

As mentioned above, one can define the below variables

18 $$\Gamma = \psi + i\phi ,\,\,\,\Pi = p + iq.$$

As a result, Eq. (17) becomes

19 $$\dot{\Gamma } + ir_{0} \Gamma = \Pi ,$$

which has the following solution

20 $$\Gamma (t) = \Gamma_{0} e^{{ - ir_{0} \,t}} + \,e^{{ - ir_{0} \,t}} I_{\Gamma } (t).$$

Here,

21 $$I_{\Gamma } (t) = \int\limits_{0}^{t} {e^{{ir_{0} \,\tau }} \,\Pi (\tau )d\tau } .$$

Hence, in order to determine the Eulerian angles, it is necessary to determine $I_{\Gamma } (t)$. By effectively employing Eqs. (13) and (18), one writes

22 $$\Pi (t) = \Bigg[ {\left( {\sqrt {\zeta_{1} } + \sqrt {\zeta_{2} } } \right)\Pi_{1} (t) + \left( {\sqrt {\zeta_{1} } - \sqrt {\zeta_{2} } } \right)\tilde{\Pi }_{1} (t)} \Bigg]/2\zeta .$$

Then, one can write $I_{\Gamma } (t)$ in the form

23 $$I_{\Gamma } (t) = \Bigg[ {(\sqrt {\zeta_{1} } + \sqrt {\zeta_{2} } )I_{1\Gamma } (t) + (\sqrt {\zeta_{1} } - \sqrt {\zeta_{2} } )I_{2\Gamma } (t)} \Bigg]/2\zeta ,$$

where

24 $$\begin{aligned} I_{1\Gamma } (t) = & \int\limits_{0}^{t} {e^{{ir_{0} \,\tau }} \,\Pi_{1} (\tau )d\tau } \\ = & \frac{{ - i\Pi_{10} }}{{{{\zeta \Bigg[r_{0} + \Bigg\{ (\lambda_{3} \, - eHl^{2} \cos \sigma )} \mathord{\left/ {\vphantom {{\zeta \Bigg[r_{0} + \Bigg\{ (\lambda_{3} \, - eHl^{2} \cos \sigma )} {\zeta_{1} D_{1} }}} \right. \kern-0pt} {\zeta_{1} D_{1} }}\Bigg\} \Bigg] + r_{0} }}\Bigg[e^{{it\Bigg\{ \zeta \Bigg[{{r_{0} + \Bigg\{ (\lambda_{3} \, - eHl^{2} \cos \sigma )} \mathord{\left/ {\vphantom {{r_{0} + \Bigg\{ (\lambda_{3} \, - eHl^{2} \cos \sigma )} {\zeta_{1} D_{1} \Bigg\} }}} \right. \kern-0pt} {\zeta_{1} D_{1} \Bigg\} }}\Bigg] + r_{0} \Bigg\} \,}} - 1\Bigg] \\ & + \frac{A}{{{{\zeta \Bigg[r_{0} + \Bigg\{ (\lambda_{3} \, - eHl^{2} \cos \sigma )} \mathord{\left/ {\vphantom {{\zeta \Bigg[r_{0} + \Bigg\{ (\lambda_{3} \, - eHl^{2} \cos \sigma )} {\zeta_{1} D_{1} }}} \right. \kern-0pt} {\zeta_{1} D_{1} }}\Bigg\} \Bigg]}}\Bigg\{ \frac{{e^{{ir_{0} \,t}} - 1}}{{r_{0} }} - \frac{1}{{{{\zeta \Bigg[r_{0} + \Bigg\{ (\lambda_{3} \, - eHl^{2} \cos \sigma )} \mathord{\left/ {\vphantom {{\zeta \Bigg[r_{0} + \Bigg\{ (\lambda_{3} \, - eHl^{2} \cos \sigma )} {\zeta_{1} D_{1} }}} \right. \kern-0pt} {\zeta_{1} D_{1} }}\Bigg\} \Bigg] + r_{0} }} \\ & \times \Bigg[e^{{it\Bigg\{ \zeta \Bigg[{{r_{0} + \Bigg\{ (\lambda_{3} \, - eHl^{2} \cos \sigma )} \mathord{\left/ {\vphantom {{r_{0} + \Bigg\{ (\lambda_{3} \, - eHl^{2} \cos \sigma )} {\zeta_{1} D_{1} \Bigg\} }}} \right. \kern-0pt} {\zeta_{1} D_{1} \Bigg\} }}\Bigg] + r_{0} \Bigg\} \,}} - 1\Bigg]\Bigg\} , \\ \end{aligned}$$

25 $$\begin{aligned} I_{2\Gamma } (t) =& \int\limits_{0}^{t} {e^{{ir_{0} \,\tau }} \,\tilde{\Pi }_{1} (\tau )d\tau } \hfill \\ =& \frac{{ - i\tilde{\Pi }_{10} }}{{r_{0} - \zeta \Bigg[{{r_{0} + \Bigg\{ (\lambda_{3} \, - eHl^{2} \cos \sigma )} \mathord{\left/ {\vphantom {{r_{0} + \Bigg\{ (\lambda_{3} \, - eHl^{2} \cos \sigma )} {\zeta_{1} D_{1} }}} \right. \kern-0pt} {\zeta_{1} D_{1} }}\Bigg\} \Bigg]}}\Bigg[e^{{it\Bigg\{ r_{0} - \zeta \Bigg[{{r_{0} + \Bigg\{ (\lambda_{3} \, - eHl^{2} \cos \sigma )} \mathord{\left/ {\vphantom {{r_{0} + \Bigg\{ (\lambda_{3} \, - eHl^{2} \cos \sigma )} {\zeta_{1} D_{1} }}} \right. \kern-0pt} {\zeta_{1} D_{1} }}\Bigg\} \Bigg]\Bigg\} \,}} - 1\Bigg] \hfill \\& - \frac{{\tilde{A}}}{{\zeta \Bigg[{{r_{0} + \Bigg\{ (\lambda_{3} \, - eHl^{2} \cos \sigma )} \mathord{\left/ {\vphantom {{r_{0} + \Bigg\{ (\lambda_{3} \, - eHl^{2} \cos \sigma )} {\zeta_{1} D_{1} }}} \right. \kern-0pt} {\zeta_{1} D_{1} }}\Bigg\} \Bigg]}}\Bigg\{ \frac{{e^{{ir_{0} \,t}} - 1}}{{r_{0} }} - \frac{1}{{r_{0} - \zeta \Bigg[{{r_{0} + \Bigg\{ (\lambda_{3} \, - eHl^{2} \cos \sigma )} \mathord{\left/ {\vphantom {{r_{0} + \Bigg\{ (\lambda_{3} \, - eHl^{2} \cos \sigma )} {\zeta_{1} D_{1} \Bigg\} }}} \right. \kern-0pt} {\zeta_{1} D_{1} \Bigg\} }}\Bigg]}} \hfill \\ & \times \Bigg[e^{{it\Bigg\{ r_{0} - \zeta \Bigg[{{r_{0} + \Bigg\{ (\lambda_{3} \, - eHl^{2} \cos \sigma )} \mathord{\left/ {\vphantom {{r_{0} + \Bigg\{ (\lambda_{3} \, - eHl^{2} \cos \sigma )} {\zeta_{1} D_{1} }}} \right. \kern-0pt} {\zeta_{1} D_{1} }}\Bigg\} \Bigg]\Bigg\} \,}} - 1\Bigg]\Bigg\} . \hfill \\ \end{aligned}$$

As a result, it is possible to express the solutions for the Eulerian angles explicitly using circular functions. These functions have limited forms as time progresses, helping to ensure that the nutation remains bounded. As a result, the solutions to Euler’s angles have the forms

26 $$\begin{aligned} \, \psi (t) = & {\text{Re}} \Bigg[\Gamma (t)\Bigg], \\ \phi (t) = & {\text{Im}} \Bigg[\Gamma (t)\Bigg], \\ \theta (t) = & r_{0} \,t + \theta_{0} . \\ \end{aligned}$$

**Figure 8:**
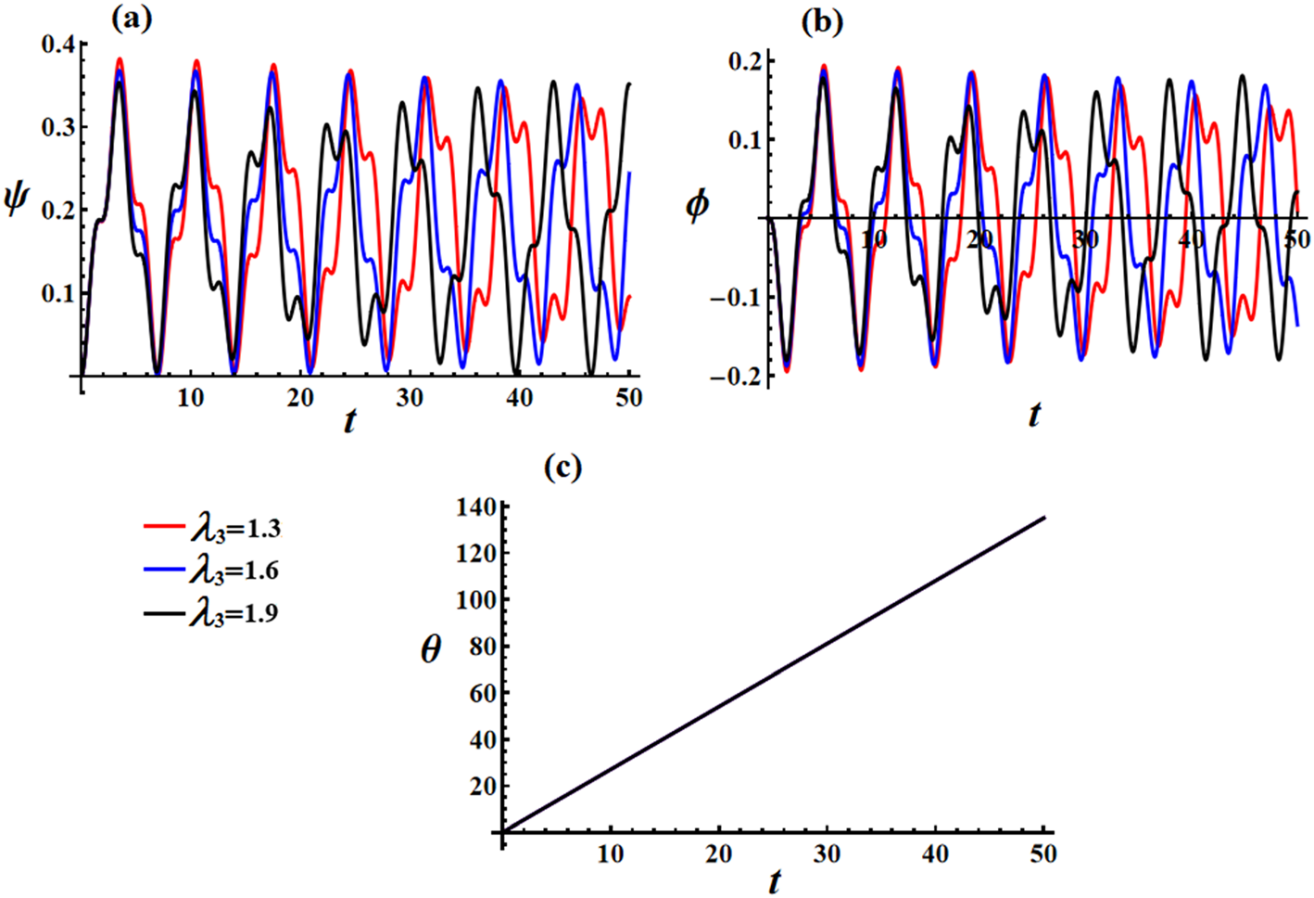
Reveals the solutions of Euler’s angles at $e = 0.003\,{\text{C,}}\,\,M_{1} = 4.0\,{\text{N}}\,{\text{m}}$ when $\lambda_{3} ( = 1.3,1.6,1.9)\,{\text{kg}}\,{\text{m}}^{2} \,{\text{s}}^{ - 1}$: (**a**) the precession angle $\psi ,$ (**b**) the proper rotation angles $\phi ,$ and (**c**) the nutation angle $\theta$.

Curves in Fig. 8 depict the graphs of Euler’s angles $\psi ,\phi ,$ and $\theta$, where they are calculated according to the aforementioned data and the mathematical form of Eq. (26). Similar to the previous illustrations, an increase in values of the GM results in quasi-periodic waves, in which their amplitudes decrease for both precession and proper rotation angles, as seen in Fig. 8a,b. However, the nutation angle remains constant, as it is directly proportional to time, as explored in Fig. 8c.

**Figure 9:**
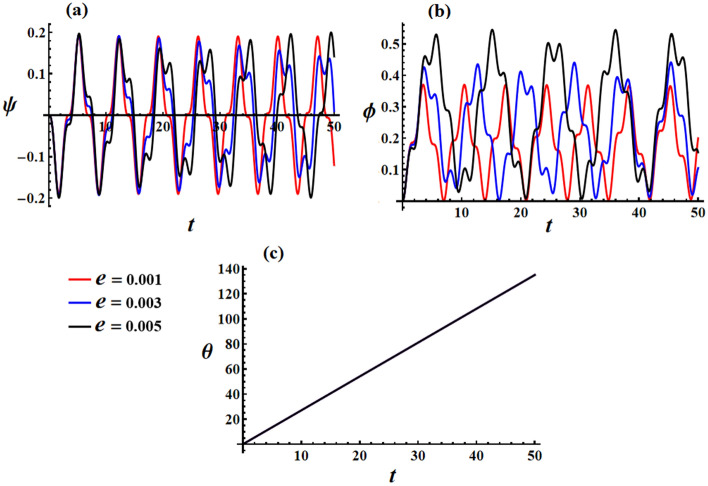
Describes time behavior of Euler’s angles: [(**a**) $\psi$, (**b**) $\phi$, and (**c**) $\theta$] at $e( = 0.001,0.003,0.005){\text{C}}$.

Just like the previous illustrations, the increase in charge’s value leads to higher precession and proper rotation angles over time, as shown in portions (a) and (b) of Fig. 9. However, it is important to note that the nutation angle remains unchanged, as depicted in Fig. 9c.

**Figure 10:**
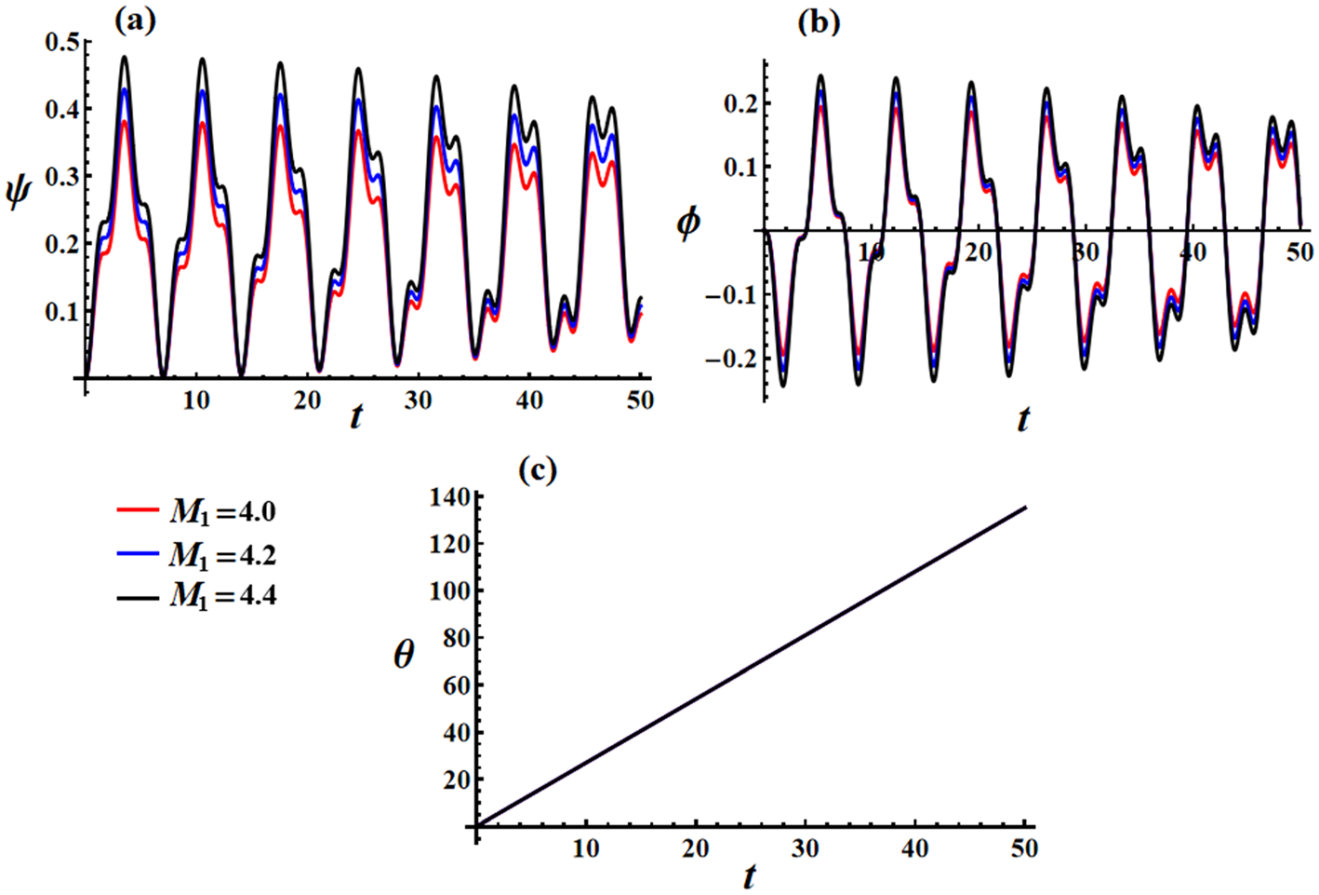
Elucidates the temporal characteristics of Euler’s angles: [(**a**) $\psi$, (**b**) $\phi$, and (**c**) $\theta$] at the values of Fig. 6.

Upon examining the graphed curves depicted in Fig. 10, one can observe the temporal fluctuations of Euler’s angles as $M_{1}$ assumes various values, as mentioned above. It becomes evident that $M_{1}$ undergoes alterations, resulting in periodic waves with slight deviations at the peaks and troughs of these waves, as depicted in both portions (a) and (b) of this figure. Additionally, a marginal enhancement in the amplitude of the plotted waves, representing precession and proper rotation angles, can be noted. In contrast, the nutation angle remains unaltered as it is directly connected to the progression of time, as explored in Fig. 10c.

### The angular momentum solution

In this subsection, we presented an approach to solving the problem, specifically concerning the angular momentum. The following equation establishes the connection between the angular momentum vector’s inertial and body components^4^

27 $$\, \Bigg[ \begin{gathered} G_{1} \hfill \\ G_{2} \hfill \\ G_{3} \hfill \\ \end{gathered} \Bigg] = \Bigg[ R \Bigg]\Bigg[ \begin{gathered} D_{1} p \hfill \\ D_{2} q \hfill \\ D_{3} r + \lambda_{3} \hfill \\ \end{gathered} \Bigg].$$

Assuming that

28 $$\, L_{1} = D_{1} p,\,\,\,\,\,\,\,L_{2} = D_{2} q,\,\,\,\,\,\,\,L_{3} = D_{3} r + \lambda_{3} .$$

The matrix $\Bigg[ R \Bigg]$, representing the direction cosines, is provided for a 3-1-2 Euler’s angle sequence^3^ as

29 $$\, \Bigg[ R \Bigg] = \Bigg[ {\begin{array}{*{20}c} {R_{11} } & {R_{12} } & {R_{13} } \\ {R_{21} } & {R_{22} } & {R_{23} } \\ {R_{31} } & {R_{32} } & {R_{33} } \\ \end{array} } \Bigg],$$

where

30 $$\begin{aligned} \, R_{11} = & \cos \theta \cos \phi - \sin \theta \sin \psi \sin \phi ,\, \\ R_{12} = & - \sin \theta \cos \psi , \\ R_{13} = & \cos \theta \sin \phi + \sin \theta \sin \psi \cos \phi , \\ R_{21} = & \sin \theta \cos \phi + \cos \theta \sin \psi \sin \phi ,\, \\ R_{22} = & \cos \theta \cos \psi , \\ R_{23} = & \cos \theta \sin \phi - \cos \theta \sin \psi \cos \phi , \\ R_{31} = & - \cos \psi \sin \phi ,\, \\ R_{32} = & \sin \psi , \\ R_{33} = & \cos \psi \cos \phi . \\ \end{aligned}$$

The transverse angular momentum vector has a complex form that is based on Eqs. (27) and (30) as

31 $$G = e^{i\theta } \Bigg[L_{1} (\cos \phi + i\sin \psi \sin \phi ) + iL_{2} \cos \psi + L_{3} (\sin \phi - i\sin \psi \cos \phi )\Bigg].$$

Now, inserting the following variables

32 $$G = G_{1} + iG_{2} ,\,\,\,L = L_{1} + iL_{2} ,$$

into (31), assuming that both $\psi$ and $\phi$ are small and their product $\psi \phi$ is insignificant, then we may write

33 $$G = e^{i\theta } \Bigg[L - i\Gamma L_{3} \Bigg].$$

The comparison between the real and imaginary components on both sides allows us to obtain the solutions for $G_{1}$ and $G_{2}$, incorporate the previously determined parameter values in (33) that are influenced by both the GM, and the electromagnetic force field, and manifest as

34 $$G_{1} = {\text{Re}} \Bigg[G\Bigg],\,\,\,\,\,\,\,\,\,G_{2} = {\text{Im}} \Bigg[G\Bigg].$$

A different approach for solving the equation is adjusted so that it no longer relies on the GM and electromagnetic force field. By substituting the values from Eqs. (18), (26), (28), and (32) into Eq. (33), and manipulating them algebraically, one can obtain another solution

35 $$G = ir_{0}^{ - 1} M(1 - e^{{i\,r_{0} \,t}} ),$$

where

36 $$M = M_{1} + iM_{2} .$$

**Figure 11:**
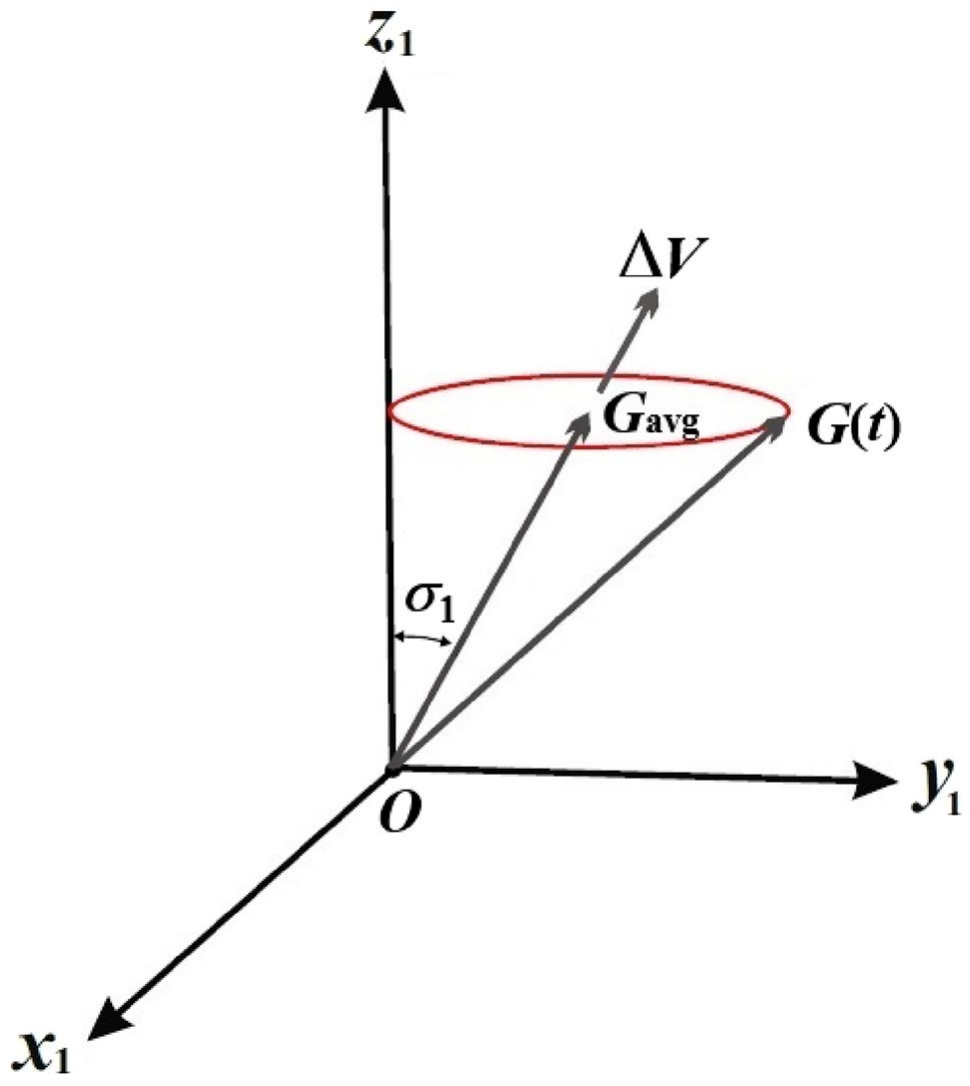
Shows the motion of angular momentum vector inside inertial space.

In the inertial plane $(G_{1} ,G_{2} )$, Eq. (36) represents a circle with a center at $( - r_{0}^{ - 1} M_{2} ,r_{0}^{ - 1} M_{1} )$ and a radius $r_{0}^{ - 1} \sqrt {M_{1}^{2} + M_{2}^{2} }$, as shown in Fig. 11, in the case where $M_{1} \ne 0$ and $M_{2} = 0$. The $G$ vector represents a small circle in inertial space, with its center representing the average position of angular momentum. The angular momentum has nonzero projections on the inertial axes $x_{1}$ and $y_{1}$, and no projection on the axis $Ox_{1}$. Assuming $\sigma_{1} \ll 1\,\,{\text{rad}}$, the average pointing error (APE) of the angular momentum vector concerning the axis $Oz_{1}$ is represented by $\sigma_{1}$.

**Figure 12:**
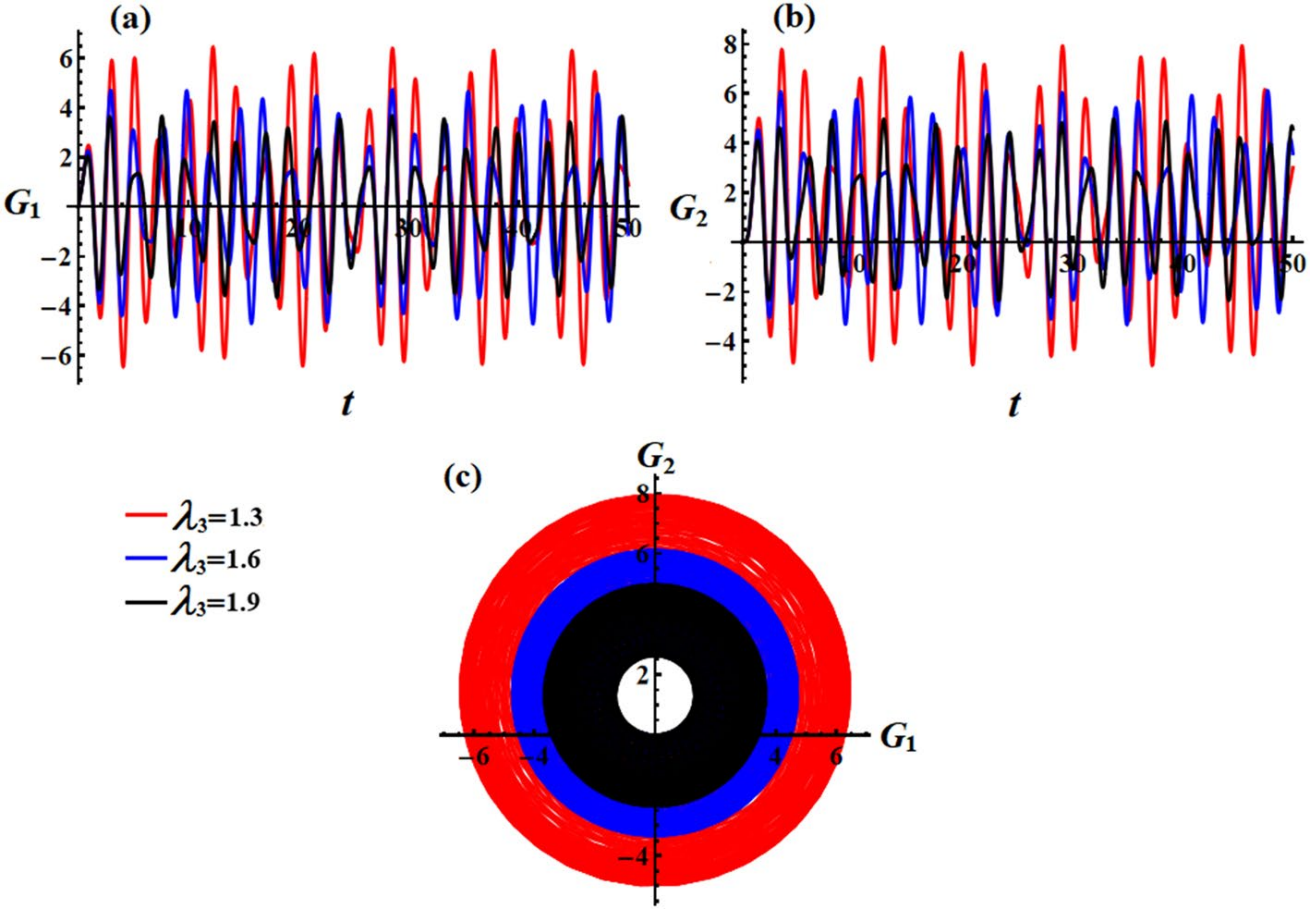
Describes the functions $G_{1} (t),\,\,G_{2} (t)$ and the corresponding curves in the plane $G_{1} G_{2}$ at $e = 0.003\,{\text{C,}}\,\,M_{1} = 4.0{\text{N m}}$ when $\lambda_{3} ( = 1.3,1.6,1.9)\,{\text{kg}}\,{\text{m}}^{2} \,{\text{s}}^{ - 1}$.

**Figure 13:**
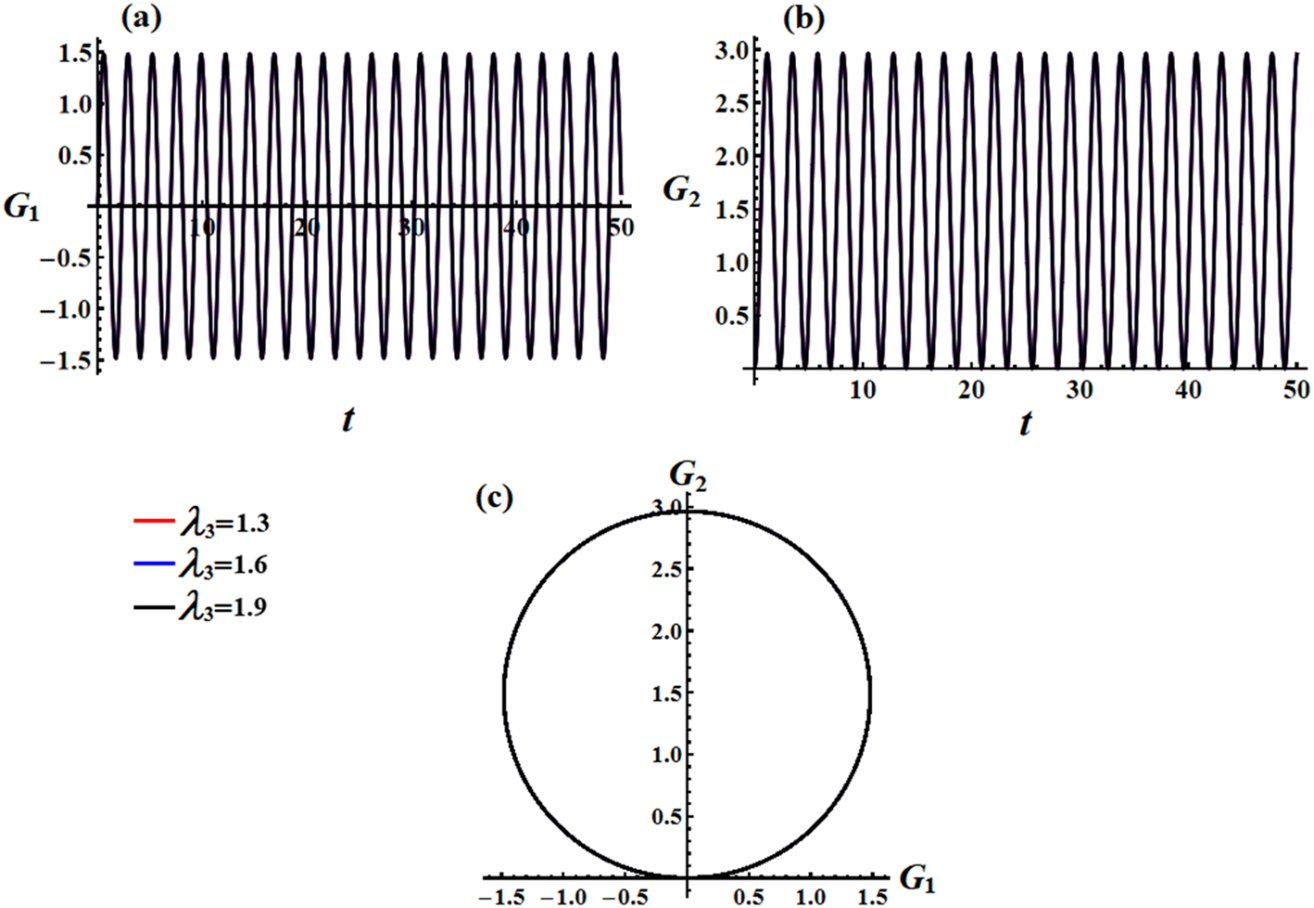
Illustrates the corresponding curves in the plane $G_{1} G_{2}$ for Figs. 6 and 7 at $e = 0.003\,{\text{C,}}\,\,M_{1} = 4.0\,{\text{N}}\,{\text{m}}$ when $\lambda_{3} ( = 1.3,1.6,1.9)\,{\text{kg}}\,{\text{m}}^{2} \,{\text{s}}^{ - 1}$.

In Figs. 12 and 13, simulations were conducted for two different scenarios regarding the angular momentum components $G_{1}$ and $G_{2}$, as in formulas (33) and (35), respectively. The first scenario considers the effect of GM’s value on the solution mentioned in Eqs. (33) and (34). It is noted that the behavior of the graphed waves of the angular momentum’s components $G_{1}$ and $G_{2}$ has decreased in their amplitudes over the time interval, as explored in Fig. 12a,b. The second scenario deals with the variation of the GM when the small approximation is applied to remove some terms in the equation, as in parts (a) and (b) of Fig. 13. The observed phenomenon in these parts reveals that the drawn waves have no variation with the change of $\lambda_{3}$ values. These waves have periodicity forms with the same amplitudes and wavelength.

The obtained AS for the angular momentum vector has been represented as phase plane curves, as in Figs. 12c and 13c. The drawn curves in Fig. 12c correspond to the curves in parts (a) and (b) of Fig. 12 that shows quasi-circular trajectories in the plane $(G_{1} ,G_{2} )$ with a center at $( - M_{2} D_{3}^{ - 1} r_{0}^{ - 2} ,M_{1} D_{3}^{ - 1} r_{0}^{ - 2} )$ and a radius $\sqrt {{{M_{1}^{2} + M_{2}^{2} } \mathord{\left/ {\vphantom {{M_{1}^{2} + M_{2}^{2} } {D_{3} r_{0}^{2} }}} \right. \kern-0pt} {D_{3} r_{0}^{2} }}}$. It’s obvious that the increase in the GM values implies a decrease in this circle radius which again implies a decrease in the trajectory quasi-paths. As Fig. 13c formulates a circle with a center at $( - M_{2} r_{0}^{ - 1} ,M_{1} r_{0}^{ - 1} )$ and a radius $r_{0}^{ - 1} \sqrt {M_{1}^{2} + M_{2}^{2} }$.

**Figure 14:**
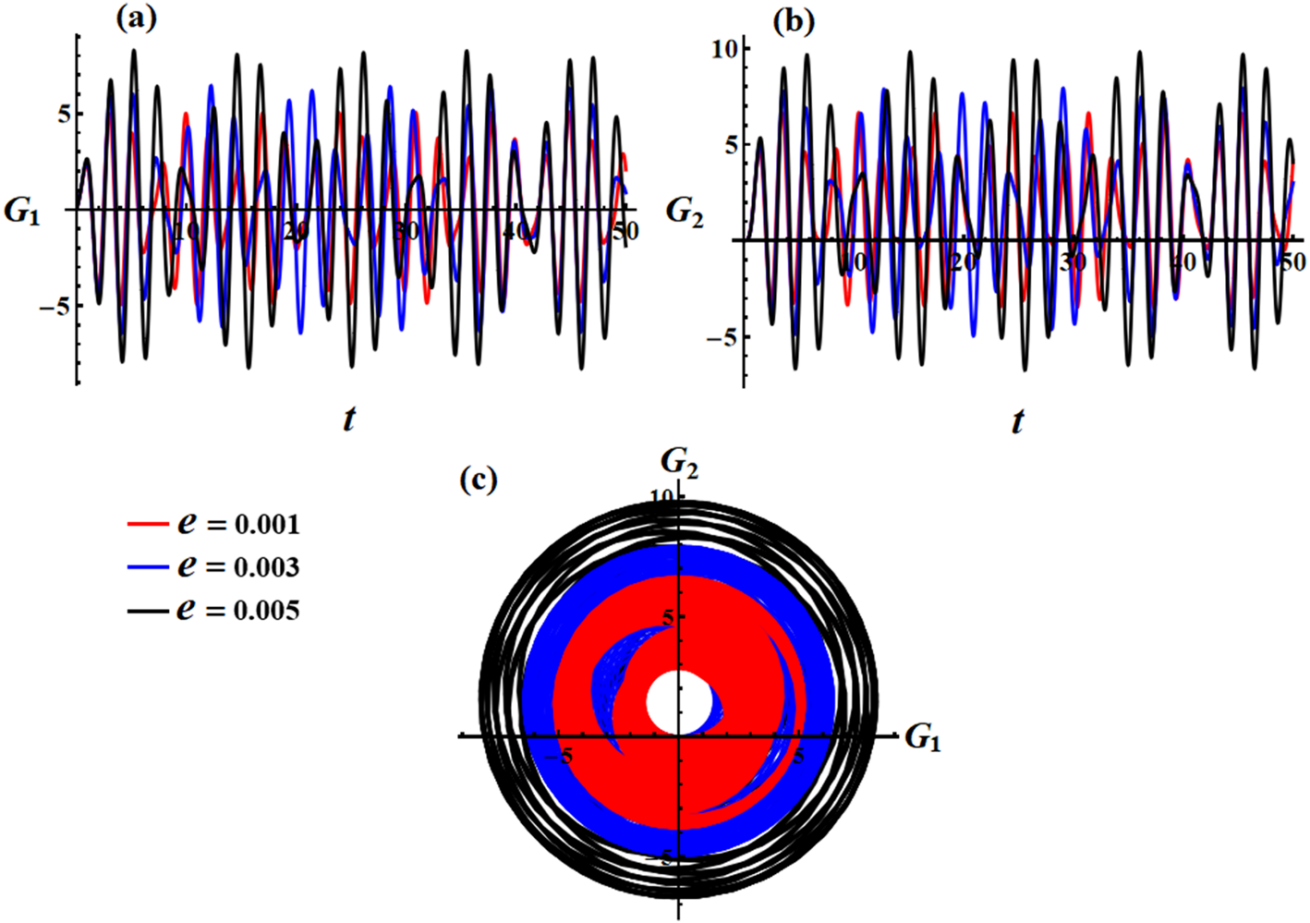
Describes the behavior of the functions $G_{1} (t)$ and $G_{2} (t)$ at $e( = 0.001,0.003,0.005){\text{C}}$ when $\lambda_{3} = 1.3\,{\text{kg}}\,{\text{m}}^{2} \,{\text{s}}^{ - 1} ,\,M_{1} = 4.0\,{\text{N}}\,{\text{m}}$.

**Figure 15:**
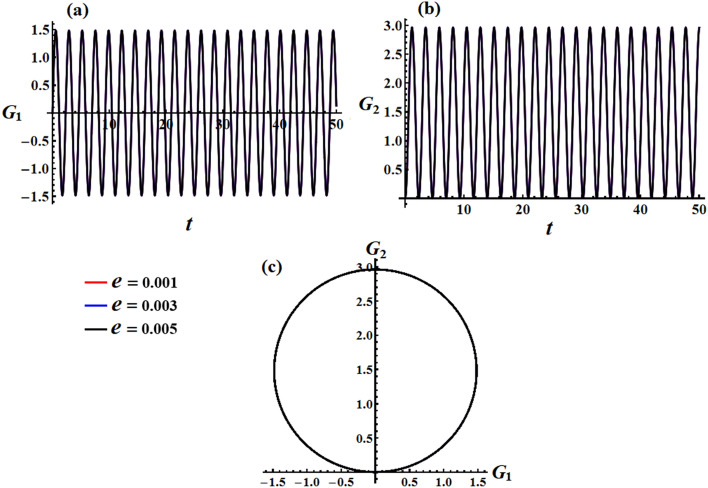
Reveals the curves of the functions $G_{1} (t),\,\,G_{2} (t)$ and in the plane $G_{1} G_{2}$ at $\lambda_{3} = 1.3\,{\text{kg}}\,{\text{m}}^{2} \,{\text{s}}^{ - 1} ,\,M_{1} = 4.0\,{\text{N}}\,{\text{m}}$ when $e( = 0.001,0.003,0.005){\text{C}}$.

Figures 14 and 15 illustrate the action of the point charge values on the behavior of the components $G_{1}$ and $G_{2}$ of the angular momentum when it is impacted by a constant value of GM and IBFT equals $\lambda_{3} = 1.3\,{\text{kg}}\,{\text{m}}^{2} \,{\text{s}}^{ - 1} {,}\,\,M_{1} = 4.0\,{\text{N}}\,{\text{m}}$. It is obvious from the included drawn curves in Fig. 14 that altering the charge’s value leads to an escalation of the angular momentum, where the fluctuation’s number remains unchanged. Conversely, there is no variation in the curves of Fig. 15 with the various values of the charge, which is due to the absence of the external forces using a small approximation in the calculations of Eq. (35).

The related diagrams of phase plane curves in the plane $G_{1} G_{2}$ for the graphs in parts (a) and (b) of Figs. 14 and 15 are presented in Figs. 14c and 15c, respectively. The observation states that the enclosed loops in part (c) of Fig. 14 display that the plotted trajectories appear almost circular. It is evident that an increase in the charge values leads to longer and wider trajectories, subsequently enlarging the radius of the contained circle. In contrast, Fig. 15c shows a circular trajectory that remains unaffected by any external factors.

**Figure 16:**
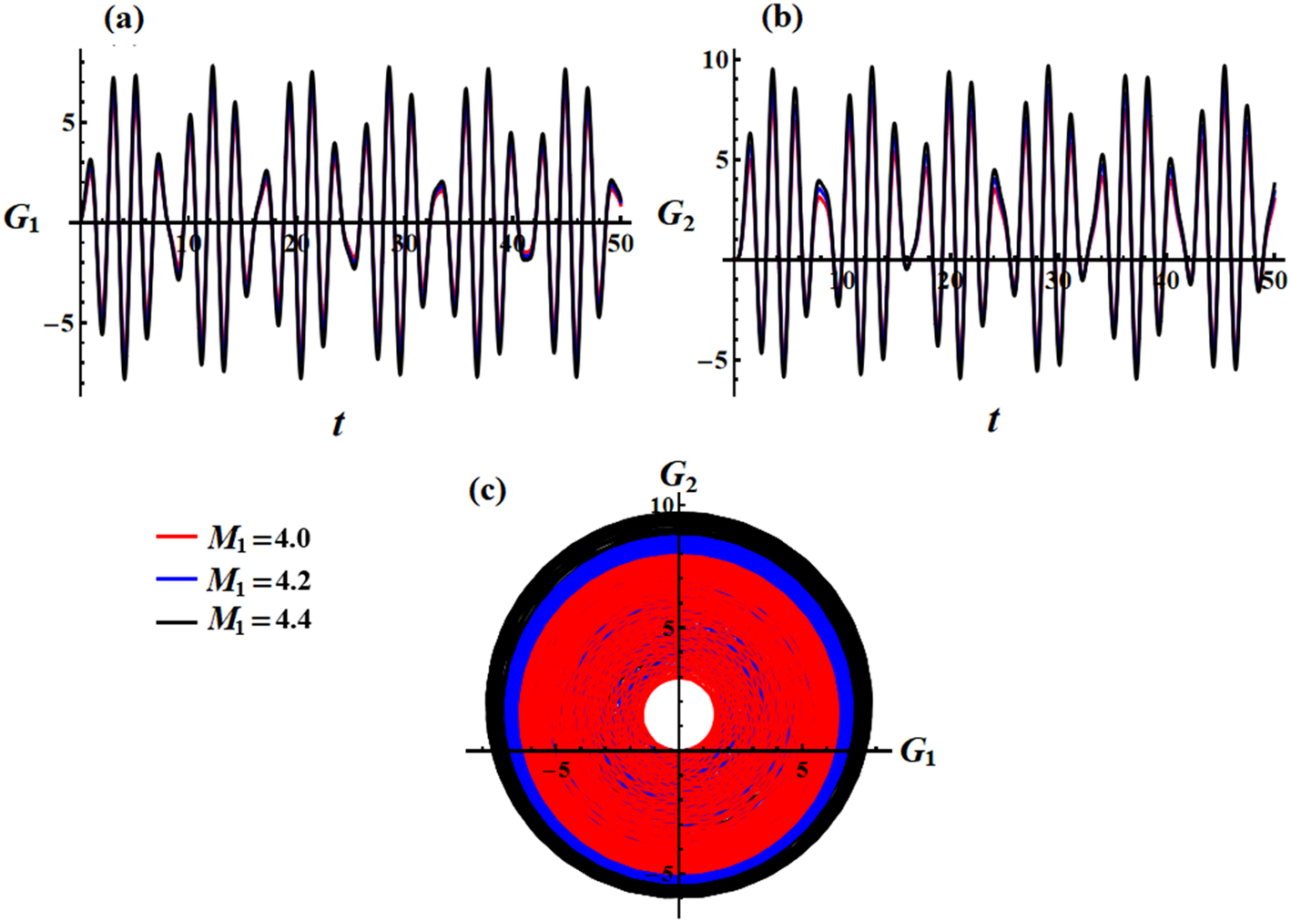
Elucidates the conduct exhibited by the functions $G_{1} (t)$ and $G_{2} (t)$ at $\lambda_{3} = 1.3\,{\text{kg}}\,{\text{m}}^{2} \,{\text{s}}^{ - 1} {,}\,\,e = 0.003{\text{C}}$ when $M_{1} ( = 4.0,4.5,5.0)\,{\text{N}}\,{\text{m}}$.

**Figure 17:**
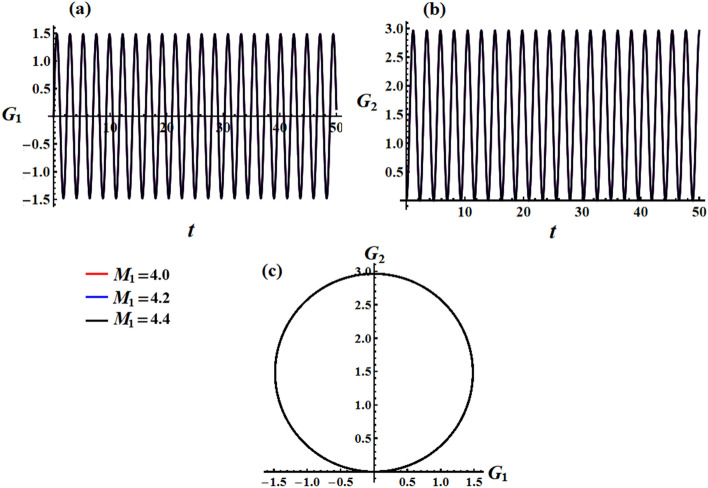
Displays the curves within the plane $G_{1} G_{2}$ at $M_{1} ( = 4.0,4.5,5.0)\,{\text{N m}}$.

The impact of $M_{1}$ on the functions $G_{1}$ and $G_{2}$ of the angular momentum can be seen in Figs. 16 and 17. By observing the plotted curves in Fig. 16, it can be deduced that altering the values of $M_{1}$ results in a marginal elevation in the amplitudes of these components. Conversely, in Fig. 17, there is no discernible fluctuation observed as $M_{1}$ varies over the investigated time period. This occurrence can be attributed to the mathematical equations governing these components (35).

The phase plane diagrams that illustrate the curves in parts (a) and (b) of Figs. 16 and 17 are depicted in Figs. 16c and 17c on the $G_{1} G_{2}$ planet, respectively. Upon closer examination, it is apparent that the loops enclosed in the last part (c) of Fig. 16 exhibit trajectories that resemble quasi-circular shapes. It becomes evident that an increase in the values of $M_{1}$ results in trajectories becoming closer. In contrast, Fig. 17c displays a circular trajectory that remains unaffected by any external factors.

### Transverse and axial velocities solutions

In the subsequent analysis, we assume that the force related to the inertial moments of the body has the components $f_{1} ,f_{2} ,$ and $f_{3}$ are constants as the applied torques to the body. The relation between the accelerations of inertia and the body analogous to Newton’s second law in rotational kinematics is expressed as follows^4^

37 $$\, \Bigg[ \begin{gathered} \dot{\omega }_{1} \hfill \\ \dot{\omega }_{2} \hfill \\ \dot{\omega }_{3} \hfill \\ \end{gathered} \Bigg] = \Bigg[ R \Bigg]\left[ \begin{gathered} f_{1} \hfill \\ f_{2} \hfill \\ f_{3} \hfill \\ \end{gathered} \right].$$

As $\psi$ and $\phi$ are small, Eq. (30) can be transformed into

38 $$\, \Bigg[ R \Bigg] = \left[ {\begin{array}{*{20}c} {\cos \theta } & { - \sin \theta } & {\phi \cos \theta + \psi \sin \theta } \\ {\sin \theta } & {\cos \theta } & {\phi \cos \theta - \psi \cos \theta } \\ { - \phi } & \psi & 1 \\ \end{array} } \right].$$

Introducing the new complex functions

39 $$\omega = \omega_{1} + i\omega_{2} ,\,\,\,f = f_{1} + if_{2} .$$

Making use of Eqs. (18) and (37) to express the transverse acceleration and the axial one as follows

40 $$\dot{\omega } = m^{ - 1} e^{i\theta } \Bigg[f - i\,\Gamma f_{3} \Bigg],$$

and

41 $$\dot{\omega }_{3} = m^{ - 1} \Bigg\{ f_{3} + {{\Bigg[i(\tilde{f}\Gamma - f\tilde{\Gamma })} \mathord{\left/ {\vphantom {{\Bigg[i(\tilde{f}\Gamma - f\tilde{\Gamma })} {2\Bigg]}}} \right. \kern-0pt} {2\Bigg]}}\Bigg\} .$$

In a later part, the solution of $\omega_{3}$ will be estimated. Referring to Eq. (40), the integration of the transverse acceleration has the form

42 $$\omega (t) = m^{ - 1} \Bigg\{ {f\int\limits_{0}^{t} {e^{i\,\theta (\tau )} \,d\tau } - if_{3} \int\limits_{0}^{t} {e^{i\,\theta (\tau )} \Gamma (\tau )\,d\tau } } \Bigg\}.$$

Substituting (16) and (18) for the values of $\theta$ and $\Gamma$ into Eq. (42) to yield

43 $$\omega (t) = \omega (0) + ie^{{i\,\theta_{0} }} m^{ - 1} \Bigg\{ f\,r_{0}^{ - 1} (1 - e^{{i\,r_{0} t}} ) - f_{3} \Bigg[\Gamma_{0} t + I_{\omega } (t)\Bigg]\Bigg\} ,$$

where

44 $$I_{\omega } (t) = {{\Bigg[(\sqrt {\zeta_{1} } + \sqrt {\zeta_{2} } )I_{1\omega } (t) + (\sqrt {\zeta_{1} } - \sqrt {\zeta_{2} } )I_{2\omega } (t)\Bigg]} \mathord{\left/ {\vphantom {{\Bigg[(\sqrt {\zeta_{1} } + \sqrt {\zeta_{2} } )I_{1\omega } (t) + (\sqrt {\zeta_{1} } - \sqrt {\zeta_{2} } )I_{2\omega } (t)\Bigg]} {2\zeta }}} \right. \kern-0pt} {2\zeta }},$$

45 $$\begin{aligned} I_{1\omega } (t) = & \int\limits_{0}^{t} {I_{1\Gamma } (\tau )d\tau } \hfill \\ = &\frac{{ - i\Pi_{10} }}{{\zeta \Bigg[{{r_{0} + \Bigg\{ (\lambda_{3} \, - eHl^{2} \cos \sigma )} \mathord{\left/ {\vphantom {{r_{0} + \Bigg\{ (\lambda_{3} \, - eHl^{2} \cos \sigma )} {\zeta_{1} D_{1} \Bigg\} }}} \right. \kern-0pt} {\zeta_{1} D_{1} \Bigg\} }}\Bigg] + r_{0} }}\Bigg[\frac{{i(1 - e^{{it\Bigg\{ \zeta \Bigg[{{r_{0} + \Bigg\{ (\lambda_{3} \, - eHl^{2} \cos \sigma )} \mathord{\left/ {\vphantom {{r_{0} + \Bigg\{ (\lambda_{3} \, - eHl^{2} \cos \sigma )} {\zeta_{1} D_{1} }}} \right. \kern-0pt} {\zeta_{1} D_{1} }}\Bigg\} \Bigg] + r_{0} \Bigg\} \,}} )}}{{\zeta \Bigg[{{r_{0} + \Bigg\{ (\lambda_{3} \, - eHl^{2} \cos \sigma )} \mathord{\left/ {\vphantom {{r_{0} + \Bigg\{ (\lambda_{3} \, - eHl^{2} \cos \sigma )} {\zeta_{1} D_{1} \Bigg\} }}} \right. \kern-0pt} {\zeta_{1} D_{1} \Bigg\} }}\Bigg] + r_{0} }} - t\Bigg] \hfill \\ &+ \frac{A}{{\zeta \Bigg[{{r_{0} + \Bigg\{ (\lambda_{3} \, - eHl^{2} \cos \sigma )} \mathord{\left/ {\vphantom {{r_{0} + \Bigg\{ (\lambda_{3} \, - eHl^{2} \cos \sigma )} {\zeta_{1} D_{1} \Bigg\} }}} \right. \kern-0pt} {\zeta_{1} D_{1} \Bigg\} }}\Bigg]}}\Bigg\{ r_{0}^{ - 1} \Bigg[\Bigg\{ ir_{0}^{ - 1} (1 - e^{{ir_{0} \,t}} ) - t\Bigg\} \hfill \\ & - \frac{1}{{\zeta \Bigg[{{r_{0} + \Bigg\{ (\lambda_{3} \, - eHl^{2} \cos \sigma )} \mathord{\left/ {\vphantom {{r_{0} + \Bigg\{ (\lambda_{3} \, - eHl^{2} \cos \sigma )} {\zeta_{1} D_{1} \Bigg\} }}} \right. \kern-0pt} {\zeta_{1} D_{1} \Bigg\} }}\Bigg] + r_{0} }}\Bigg[\frac{{i(1 - e^{{it\Bigg\{ \zeta \Bigg[{{r_{0} + \Bigg\{ (\lambda_{3} \, - eHl^{2} \cos \sigma )} \mathord{\left/ {\vphantom {{r_{0} + \Bigg\{ (\lambda_{3} \, - eHl^{2} \cos \sigma )} {\zeta_{1} D_{1} }}} \right. \kern-0pt} {\zeta_{1} D_{1} }}\Bigg\} \Bigg] + r_{0} \Bigg\} \,}} )}}{{\zeta \Bigg[{{r_{0} + \Bigg\{ (\lambda_{3} \, - eHl^{2} \cos \sigma )} \mathord{\left/ {\vphantom {{r_{0} + \Bigg\{ (\lambda_{3} \, - eHl^{2} \cos \sigma )} {\zeta_{1} D_{1} \Bigg\} }}} \right. \kern-0pt} {\zeta_{1} D_{1} \Bigg\} }}\Bigg] + r_{0} }} - t\Bigg]\Bigg\} , \hfill \\ \end{aligned}$$

46 $$\begin{aligned} I_{2\omega } (t) =& \int\limits_{0}^{t} {I_{2\Gamma } (\tau )d\tau } \hfill \\ = & \frac{{ - i\tilde{\Pi }_{10} }}{{r_{0} - \zeta \Bigg[{{r_{0} + \Bigg\{ (\lambda_{3} \, - eHl^{2} \cos \sigma )} \mathord{\left/ {\vphantom {{r_{0} + \Bigg\{ (\lambda_{3} \, - eHl^{2} \cos \sigma )} {\zeta_{1} D_{1} \Bigg\} }}} \right. \kern-0pt} {\zeta_{1} D_{1} \Bigg\} }}\Bigg]}}\Bigg[\frac{{i(e^{{it\Bigg\{ r_{0} - \zeta \Bigg[r_{0} + \Bigg\{ {{\zeta (\lambda_{3} \, - eHl^{2} \cos \sigma )} \mathord{\left/ {\vphantom {{\zeta (\lambda_{3} \, - eHl^{2} \cos \sigma )} {\zeta_{1} D_{1} \Bigg\} }}} \right. \kern-0pt} {\zeta_{1} D_{1} \Bigg\} }}\Bigg]\Bigg\} }} - 1)}}{{r_{0} - \zeta \Bigg[{{r_{0} + \Bigg\{ (\lambda_{3} \, - eHl^{2} \cos \sigma )} \mathord{\left/ {\vphantom {{r_{0} + \Bigg\{ (\lambda_{3} \, - eHl^{2} \cos \sigma )} {\zeta_{1} D_{1} }}} \right. \kern-0pt} {\zeta_{1} D_{1} }}\Bigg\} \Bigg]}} - t\Bigg] \hfill \\ & - \frac{{\tilde{A}}}{{\zeta \Bigg[{{r_{0} + \Bigg\{ (\lambda_{3} \, - eHl^{2} \cos \sigma )} \mathord{\left/ {\vphantom {{r_{0} + \Bigg\{ (\lambda_{3} \, - eHl^{2} \cos \sigma )} {\zeta_{1} D_{1} \Bigg\} }}} \right. \kern-0pt} {\zeta_{1} D_{1} \Bigg\} }}\Bigg]}}\Bigg\{ r_{0}^{ - 1} \Bigg[ir_{0}^{ - 1} (1 - e^{{ir_{0} \,t}} ) - t\Bigg] \hfill \\ & - \frac{1}{{r_{0} - \zeta \Bigg[{{r_{0} + \Bigg\{ (\lambda_{3} \, - eHl^{2} \cos \sigma )} \mathord{\left/ {\vphantom {{r_{0} + \Bigg\{ (\lambda_{3} \, - eHl^{2} \cos \sigma )} {\zeta_{1} D_{1} }}} \right. \kern-0pt} {\zeta_{1} D_{1} }}\Bigg\} \Bigg]}}\Bigg[\frac{{i(e^{{it\Bigg\{ r_{0} - \zeta \Bigg[r_{0} + \Bigg\{ {{\zeta (\lambda_{3} \, - eHl^{2} \cos \sigma )} \mathord{\left/ {\vphantom {{\zeta (\lambda_{3} \, - eHl^{2} \cos \sigma )} {\zeta_{1} D_{1} \Bigg\} }}} \right. \kern-0pt} {\zeta_{1} D_{1} \Bigg\} }}\Bigg]\Bigg\} }} - 1)}}{{r_{0} - \zeta \Bigg[{{r_{0} + \Bigg\{ (\lambda_{3} \, - eHl^{2} \cos \sigma )} \mathord{\left/ {\vphantom {{r_{0} + \Bigg\{ (\lambda_{3} \, - eHl^{2} \cos \sigma )} {\zeta_{1} D_{1} }}} \right. \kern-0pt} {\zeta_{1} D_{1} }}\Bigg\} \Bigg]}} - t\Bigg]\Bigg\} . \hfill \\ \end{aligned}$$

Equations (45) and (46) highlight the occurrence of secular terms, which are terms that consistently increase with time. The growth of the axial component of velocity $\omega_{3}$ with time is anticipated to be linear. Therefore, it is reasonable to expect secular effects in the transverse components as well. In the forthcoming section, we will delve deeper into this particular behavior, specifically concentrating on the axial velocity.

Now, we obtain the axial velocity’s solution. To achieve this objective, the integration of the axial acceleration Eq. (41) produces this solution, as follows

47 $$\omega_{3} (t) = \omega_{3} (0) + m^{ - 1} f_{3} t + \frac{i}{2m}\Bigg[\tilde{f}\int\limits_{o}^{t} {\Gamma (\tau )d\tau } - f\int\limits_{o}^{t} {\tilde{\Gamma }(\tau )d\tau } \Bigg],$$

where

48 $$\begin{aligned} \int\limits_{0}^{t} {\Gamma (\tau )d\tau } =& \int\limits_{0}^{t} {\Bigg[\Gamma_{0} e^{{ - ir_{0} \,\tau }} + \,e^{{ - ir_{0} \,\tau }} I_{\Gamma } (\tau )\Bigg]d\tau } \hfill \\ =& i\Gamma_{0} r_{0}^{ - 1} (e^{{ - ir_{0} \,t}} - 1) + I_{3\omega } (t), \hfill \\ \end{aligned}$$

and

49 $$I_{3\omega } (t) = {{\Bigg[(\sqrt {\zeta_{1} } + \sqrt {\zeta_{2} } )I_{4\omega } (t) + (\sqrt {\zeta_{1} } - \sqrt {\zeta_{2} } )I_{5\omega } (t)\Bigg]} \mathord{\left/ {\vphantom {{\Bigg[(\sqrt {\zeta_{1} } + \sqrt {\zeta_{2} } )I_{4\omega } (t) + (\sqrt {\zeta_{1} } - \sqrt {\zeta_{2} } )I_{5\omega } (t)\Bigg]} {2\zeta }}} \right. \kern-0pt} {2\zeta }},$$

50 $$\begin{aligned} I_{4\omega } (t) =& \frac{{ - i\Pi_{10} }}{{\zeta \Bigg[{{r_{0} + \Bigg\{ (\lambda_{3} \, - eHl^{2} \cos \sigma )} \mathord{\left/ {\vphantom {{r_{0} + \Bigg\{ (\lambda_{3} \, - eHl^{2} \cos \sigma )} {\zeta_{1} D_{1} }}} \right. \kern-0pt} {\zeta_{1} D_{1} }}\Bigg\} \Bigg] + r_{0} }}\Bigg\{ \frac{{i\Bigg[e^{{it\Bigg\{ {{\zeta \Bigg[r_{0} + \Bigg\{ (\lambda_{3} \, - eHl^{2} \cos \sigma )} \mathord{\left/ {\vphantom {{\zeta \Bigg[r_{0} + \Bigg\{ (\lambda_{3} \, - eHl^{2} \cos \sigma )} {\zeta_{1} D_{1} }}} \right. \kern-0pt} {\zeta_{1} D_{1} }}\Bigg\} \Bigg] + r_{0} \Bigg\} \,}} - 1\Bigg]}}{{\zeta \Bigg[{{r_{0} + \Bigg\{ (\lambda_{3} \, - eHl^{2} \cos \sigma )} \mathord{\left/ {\vphantom {{r_{0} + \Bigg\{ (\lambda_{3} \, - eHl^{2} \cos \sigma )} {\zeta_{1} D_{1} }}} \right. \kern-0pt} {\zeta_{1} D_{1} }}\Bigg\} \Bigg] + r_{0} }} - t\Bigg\} \hfill \\ & + \frac{A}{{\zeta \Bigg[{{r_{0} + \Bigg\{ (\lambda_{3} \, - eHl^{2} \cos \sigma )} \mathord{\left/ {\vphantom {{r_{0} + \Bigg\{ (\lambda_{3} \, - eHl^{2} \cos \sigma )} {\zeta_{1} D_{1} }}} \right. \kern-0pt} {\zeta_{1} D_{1} }}\Bigg\} \Bigg]}}\Bigg\{ r_{0}^{ - 1} \Bigg[ir_{0}^{ - 1} (1 - e^{{ir_{0} \,t}} ) - t\Bigg] \hfill \\ & - \frac{1}{{\zeta \Bigg[{{r_{0} + \Bigg\{ (\lambda_{3} \, - eHl^{2} \cos \sigma )} \mathord{\left/ {\vphantom {{r_{0} + \Bigg\{ (\lambda_{3} \, - eHl^{2} \cos \sigma )} {\zeta_{1} D_{1} }}} \right. \kern-0pt} {\zeta_{1} D_{1} }}\Bigg\} \Bigg] + r_{0} }}\Bigg[\frac{{i(e^{{it\Bigg\{ {{\zeta \Bigg[r_{0} + \Bigg\{ (\lambda_{3} \, - eHl^{2} \cos \sigma )} \mathord{\left/ {\vphantom {{\zeta \Bigg[r_{0} + \Bigg\{ (\lambda_{3} \, - eHl^{2} \cos \sigma )} {\zeta_{1} D_{1} }}} \right. \kern-0pt} {\zeta_{1} D_{1} }}\Bigg\} \Bigg] + r_{0} \Bigg\} \,}} - 1)}}{{\zeta \Bigg[{{r_{0} + \Bigg\{ (\lambda_{3} \, - eHl^{2} \cos \sigma )} \mathord{\left/ {\vphantom {{r_{0} + \Bigg\{ (\lambda_{3} \, - eHl^{2} \cos \sigma )} {\zeta_{1} D_{1} }}} \right. \kern-0pt} {\zeta_{1} D_{1} }}\Bigg\} \Bigg] + r_{0} }} - t\Bigg]\Bigg\} , \hfill \\ \end{aligned}$$

51 $$\begin{aligned} I_{5\omega } (t) =& \frac{{ - i\tilde{\Pi }_{10} }}{{r_{0} - \zeta \Bigg[{{r_{0} + \Bigg\{ (\lambda_{3} \, - eHl^{2} \cos \sigma )} \mathord{\left/ {\vphantom {{r_{0} + \Bigg\{ (\lambda_{3} \, - eHl^{2} \cos \sigma )} {\zeta_{1} D_{1} \Bigg\} }}} \right. \kern-0pt} {\zeta_{1} D_{1} \Bigg\} }}\Bigg]}}\Bigg\{ \frac{{i\Bigg[1 - e^{{it\Bigg\{ r_{0} - \zeta \Bigg[{{r_{0} + \Bigg\{ (\lambda_{3} \, - eHl^{2} \cos \sigma )} \mathord{\left/ {\vphantom {{r_{0} + \Bigg\{ (\lambda_{3} \, - eHl^{2} \cos \sigma )} {\zeta_{1} D_{1} \Bigg\} }}} \right. \kern-0pt} {\zeta_{1} D_{1} \Bigg\} }}\Bigg]\Bigg\} }} \Bigg]}}{{r_{0} - \zeta \Bigg[{{r_{0} + \Bigg\{ (\lambda_{3} \, - eHl^{2} \cos \sigma )} \mathord{\left/ {\vphantom {{r_{0} + \Bigg\{ (\lambda_{3} \, - eHl^{2} \cos \sigma )} {\zeta_{1} D_{1} \Bigg\} }}} \right. \kern-0pt} {\zeta_{1} D_{1} \Bigg\} }}\Bigg]}} - t\Bigg\} \hfill \\ & - \frac{{\tilde{A}}}{{\zeta \Bigg[{{r_{0} + \Bigg\{ (\lambda_{3} \, - eHl^{2} \cos \sigma )} \mathord{\left/ {\vphantom {{r_{0} + \Bigg\{ (\lambda_{3} \, - eHl^{2} \cos \sigma )} {\zeta_{1} D_{1} \Bigg\} }}} \right. \kern-0pt} {\zeta_{1} D_{1} \Bigg\} }}\Bigg]}}\Bigg\{ r_{0}^{ - 1} \Bigg[ir_{0}^{ - 1} (1 - e^{{ir_{0} \,t}} ) - t\Bigg] \hfill \\ & - \frac{1}{{r_{0} - \zeta \Bigg[{{r_{0} + \Bigg\{ (\lambda_{3} \, - eHl^{2} \cos \sigma )} \mathord{\left/ {\vphantom {{r_{0} + \Bigg\{ (\lambda_{3} \, - eHl^{2} \cos \sigma )} {\zeta_{1} D_{1} \Bigg\} }}} \right. \kern-0pt} {\zeta_{1} D_{1} \Bigg\} }}\Bigg]}}\Bigg[\frac{{i(1 - e^{{it\Bigg\{ r_{0} - \zeta \Bigg[{{r_{0} + \Bigg\{ (\lambda_{3} \, - eHl^{2} \cos \sigma )} \mathord{\left/ {\vphantom {{r_{0} + \Bigg\{ (\lambda_{3} \, - eHl^{2} \cos \sigma )} {\zeta_{1} D_{1} \Bigg\} }}} \right. \kern-0pt} {\zeta_{1} D_{1} \Bigg\} }}\Bigg]\Bigg\} }} )}}{{r_{0} - \zeta \Bigg[{{r_{0} + \Bigg\{ (\lambda_{3} \, - eHl^{2} \cos \sigma )} \mathord{\left/ {\vphantom {{r_{0} + \Bigg\{ (\lambda_{3} \, - eHl^{2} \cos \sigma )} {\zeta_{1} D_{1} \Bigg\} }}} \right. \kern-0pt} {\zeta_{1} D_{1} \Bigg\} }}\Bigg]}} - t\Bigg]\Bigg\} . \hfill \\ \end{aligned}$$

The dominant effect in Eq. (47) is represented by the expected term ${{f_{3} t} \mathord{\left/ {\vphantom {{f_{3} t} m}} \right. \kern-0pt} m}$ when $f_{3}$ is not equal to zero. Now, the significant impact of secular terms in the transverse velocities and axial velocity solutions will be examined. By utilizing Eqs. (43)–(51) and assuming all initial conditions are zero, it can be demonstrated that as the time approaches infinity, the velocity ratio is determined by

52 $$\frac{{\omega_{secular} }}{{\omega_{3secular} }} = \frac{{\omega_{1secular} }}{{\omega_{3secular} }} + i\frac{{\omega_{2secular} }}{{\omega_{3secular} }},$$

where

53 $$\frac{{\omega_{1secular} }}{{\omega_{3secular} }} = - \frac{{{{M_{2} } \mathord{\left/ {\vphantom {{M_{2} } {D_{3} r_{0}^{2} }}} \right. \kern-0pt} {D_{3} r_{0}^{2} }}}}{{\Bigg[1 + \frac{{M_{1} (f_{2} /f_{3} )}}{{\zeta_{1} D_{1} r_{0}^{2} }} - \frac{{M_{2} (f_{1} /f_{3} )}}{{\zeta_{2} D_{2} r_{0}^{2} }}\Bigg]}},$$

54 $$\frac{{\omega_{2secular} }}{{\omega_{3secular} }} = \frac{{{{M_{1} } \mathord{\left/ {\vphantom {{M_{1} } {D_{3} r_{0}^{2} }}} \right. \kern-0pt} {D_{3} r_{0}^{2} }}}}{{\Bigg[1 + \frac{{M_{1} (f_{2} /f_{3} )}}{{\zeta_{1} D_{1} r_{0}^{2} }} - \frac{{M_{2} (f_{1} /f_{3} )}}{{\zeta_{2} D_{2} r_{0}^{2} }}\Bigg]}}.$$

The observation indicates that in the absence of transverse forces, the velocity coincides with the average angular momentum vector, depicted in Fig. 11. In the absence of transverse torque, the error in velocity alignment decreases towards zero over time. However, in real-world scenarios, transverse torques generally occur due to the offset of the center of mass and misalignment of the axial thruster.

**Figure 18:**
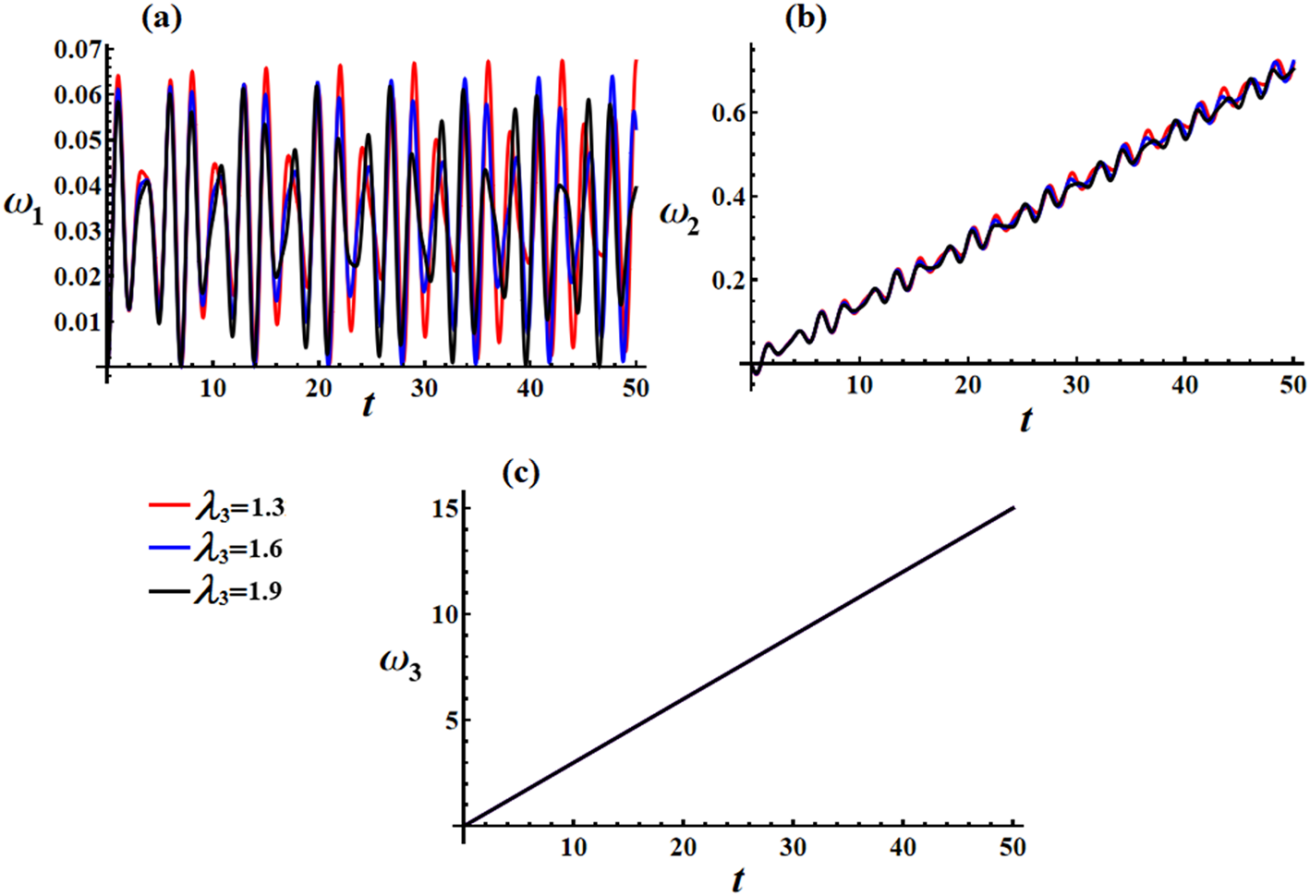
Simulates the solution of the transverse and axial velocities at $e = 0.003\,{\text{C,}}\,\,M_{1} = 4.0\,{\text{N}}\,{\text{m}}$ when $\lambda_{3} ( = 1.3,1.6,1.9)\,{\text{kg}}\,{\text{m}}^{2} \,{\text{s}}^{ - 1}$ for: (**a**) $\omega_{1}$, (**b**) $\omega_{2}$, and (**c**) $\omega_{3}$.

Figure 18 displays the AS for the transverse velocities $\omega_{1}$ and $\omega_{2}$, which are obtained by considering the real and imaginary components of Eq. (43). Equations (52)–(54) provide information about the behavior of the transverse velocities’ components. As Eq. (54) suggests when $M_{2}$ equals zero, as time approaches infinity, the ratio ${{\omega_{1secular} } \mathord{\left/ {\vphantom {{\omega_{1secular} } {\omega_{3secular} }}} \right. \kern-0pt} {\omega_{3secular} }}$ tends towards zero. It must be mentioned that the value of $\omega_{1}$ remains bounded over time, as demonstrated in Fig. 18. On the other hand, portion (c) of Fig. 18 shows that $\omega_{3}$ increases linearly as time progresses. However, due to $M_{1}$ being zero, Eq. (54) indicates that APE has a nonzero value.

**Figure 19:**
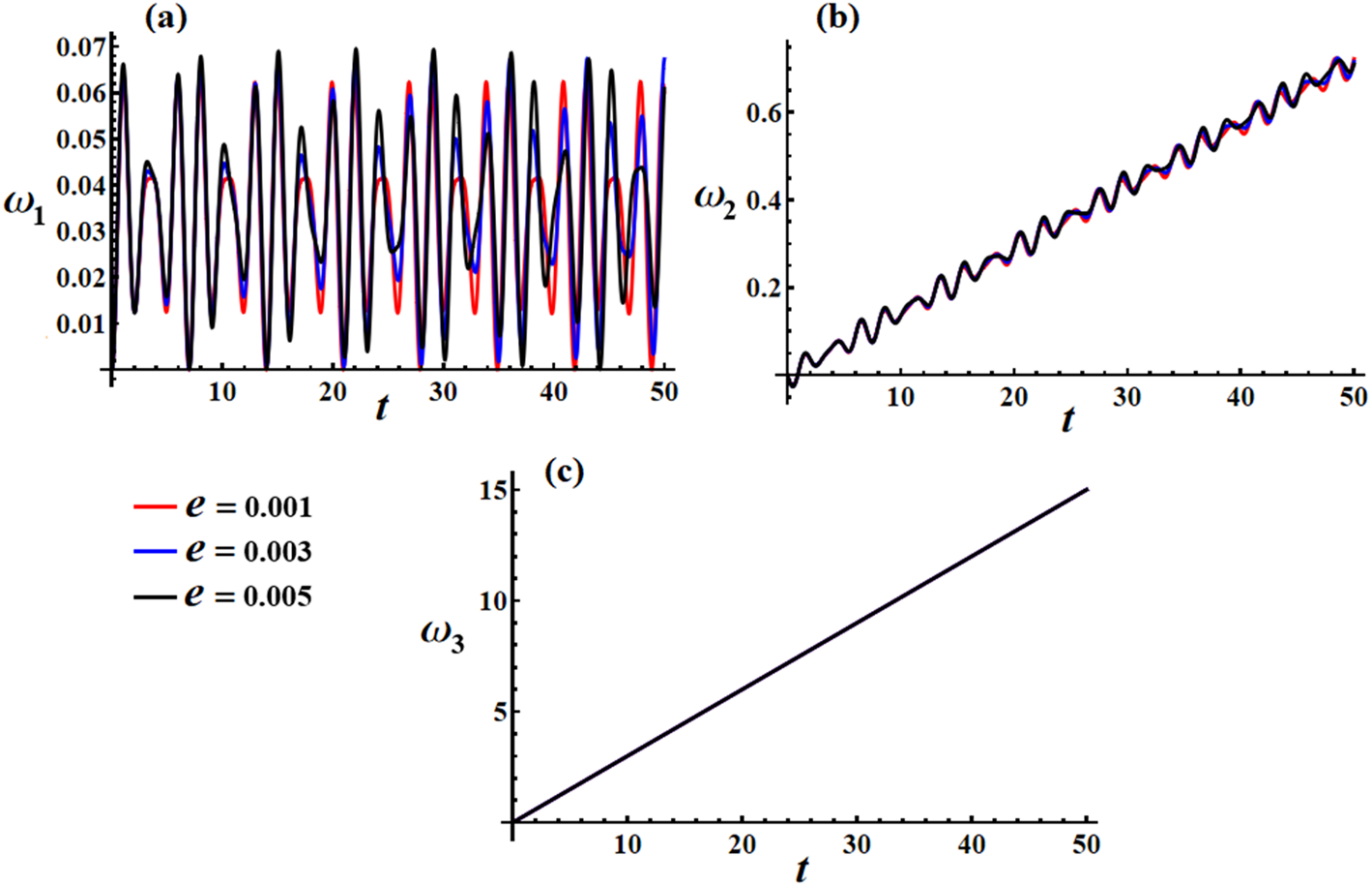
Simulates the solution of the transverse and axial velocities at $\lambda_{3} = 1.3\,{\text{kg}}\,{\text{m}}^{2} \,{\text{s}}^{ - 1} ,\,M_{1} = 4.0\,{\text{N}}\,{\text{m}}$ when $e( = 0.001,0.003,0.005){\text{C}}$ for: (**a**) $\omega_{1}$, (**b**) $\omega_{2}$, and (**c**) $\omega_{3}$.

In Fig. 19, we can observe the graphical representation of the response curves corresponding to various values of the charge $e$ on the behavior of the transverse velocities $\omega_{1}$ and $\omega_{2}$ at $\lambda_{3} = 1.3\,{\text{kg}}\,{\text{m}}^{2} \,{\text{s}}^{ - 1} ,\,M_{1} = 4.0\,{\text{N}}\,{\text{m}}$. It is worth mentioning that the increase in the charge’s value leads to an increase in both the amplitude and frequency of the $\omega_{1}$ wave, while the wavelength decreases. As for $\omega_{2}$, increasing the charge’s value results in a boost in the linear growth of $\omega_{2}$ over whole the examined time interval. It is important to note that the change of the charge’s value has no positive action on the axial angular velocity behavior, as seen in Fig. 19c.

**Figure 20:**
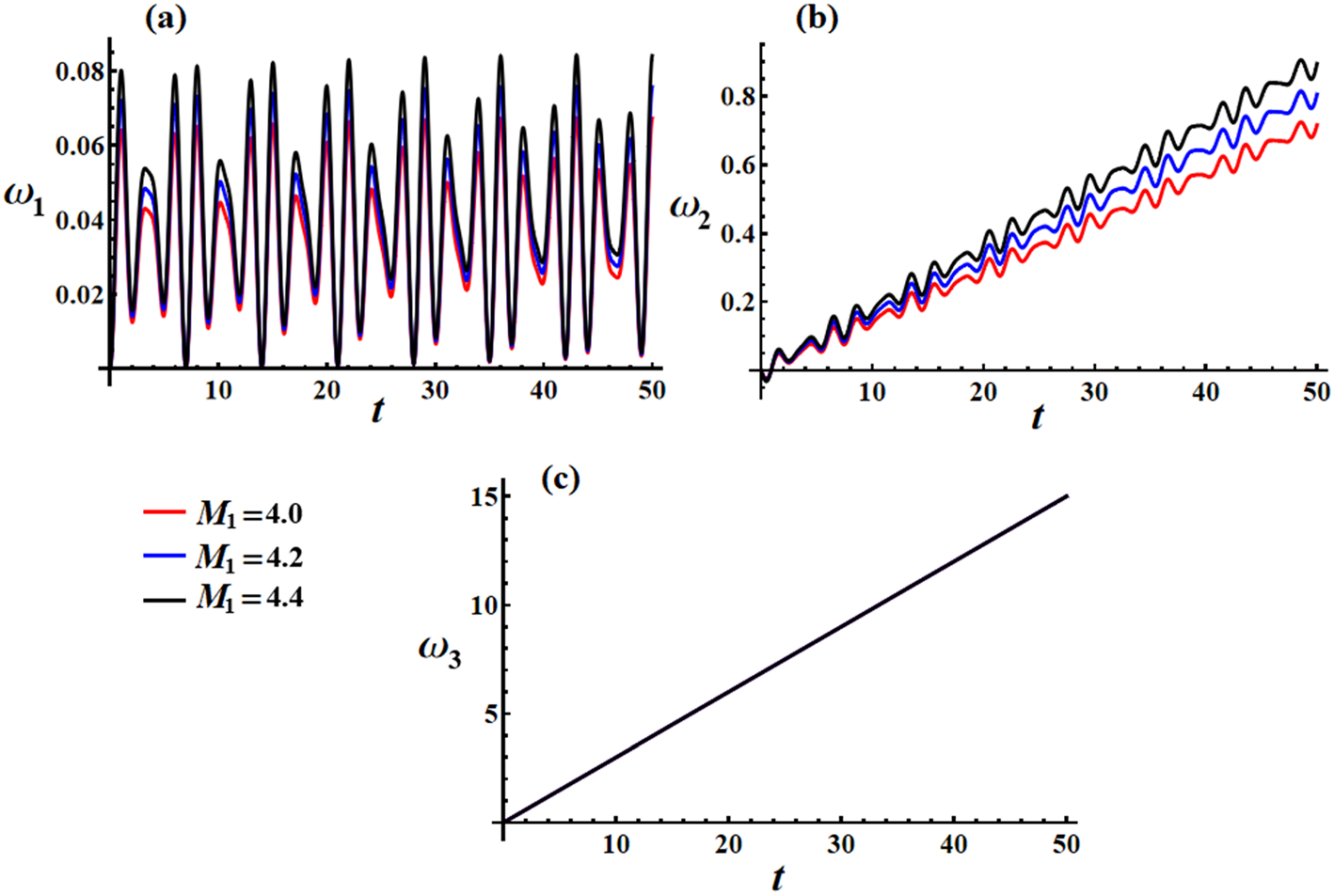
Discusses the solution of the transverse and axial velocities at $\lambda_{3} = 1.3\,{\text{kg}}\,{\text{m}}^{2} \,{\text{s}}^{ - 1} {,}\,\,e = 0.003{\text{C}}$ when $M_{1} ( = 4.0,4.5,5.0)\,{\text{N}}\,{\text{m}}$ for: (**a**) $\omega_{1}$, (**b**) $\omega_{2}$, and (**c**) $\omega_{3}$.

The curves depicted in Fig. 20 illustrate the impact of different $M_{1}$ values on the behavior of transverse velocities $\omega_{1}$ and $\omega_{2}$, with $\lambda_{3} = 1.3\,{\text{kg}}\,{\text{m}}^{2} \,{\text{s}}^{ - 1} ,\,e = 0.003{\text{C}}$. When $M_{1}$ values are increased, there is a minimal deviation observed in the amplitude of the wave representing $\omega_{1}$. However, the wavelength and frequency remain constant, as depicted in Fig. 20a. On the other hand, Fig. 20b illustrates the positive influence of $M_{1}$ values on the behavior of $\omega_{2}$, with slight fluctuations observed over the given time interval. As mentioned previously, the axial velocity $\omega_{3}$ exhibits a continuous increase over time without any variation in response to changes in $M_{1}$ values, as shown in Fig. 20c.

**Figure 21:**
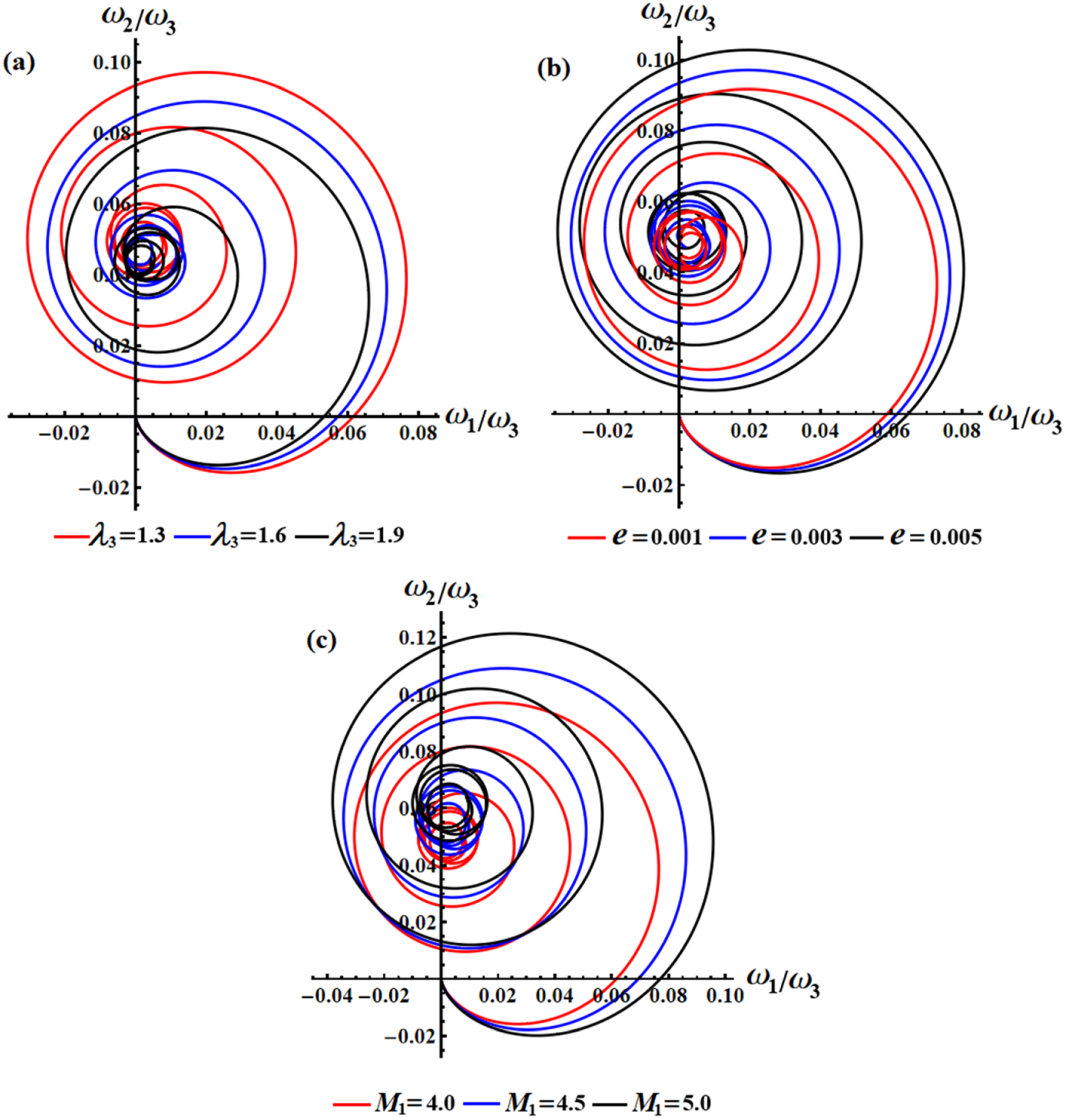
Presents the impact of $\lambda_{3} ,\,\,e,$ and $M_{1}$ values on the inertial velocity pointing $({{\omega_{1} } \mathord{\left/ {\vphantom {{\omega_{1} } {\omega_{3} }}} \right. \kern-0pt} {\omega_{3} }},{{\omega_{2} } \mathord{\left/ {\vphantom {{\omega_{2} } {\omega_{3} }}} \right. \kern-0pt} {\omega_{3} }})$.

Additionally, Fig. 21a indicates that $\omega_{2}$ also grows linearly with time, similar to $\omega_{3}$, which aligns with the representation in Fig. 11, where velocity components along the $y_{1}$ and $z_{1}$ inertial directions increase while $x_{1}$ inertial direction remains unchanged.

Figure 21a illustrates the velocity pointing in inertial space $({{\omega_{1} } \mathord{\left/ {\vphantom {{\omega_{1} } {\omega_{3} }}} \right. \kern-0pt} {\omega_{3} }},{{\omega_{2} } \mathord{\left/ {\vphantom {{\omega_{2} } {\omega_{3} }}} \right. \kern-0pt} {\omega_{3} }})$ for different values of the GM. It is evident that the velocity gradually aligns with the average path of the angular momentum vector centered at $( - M_{2} D_{3}^{ - 1} r_{0}^{ - 2} ,M_{1} D_{3}^{ - 1} r_{0}^{ - 2} )$. The effect of increasing the GM implies a decrease in $\omega_{1}$ wave amplitude while wavelength and frequency stay constant. For $\omega_{2}$, the increase in the GM value results in a decrease in the linear growth for the $\omega_{2}$ values. The variation of the GM value does not affect values as it is expressed as a linear function in time.

The plotted curves in Fig. 21b demonstrate the favorable effect of various charge values on the velocity in inertial space $({{\omega_{1} } \mathord{\left/ {\vphantom {{\omega_{1} } {\omega_{3} }}} \right. \kern-0pt} {\omega_{3} }},{{\omega_{2} } \mathord{\left/ {\vphantom {{\omega_{2} } {\omega_{3} }}} \right. \kern-0pt} {\omega_{3} }})$. The drawn curves have spiral forms, which go toward a fixed point after a specific time. It is indicated that an increase in $e$ value can cause a longer period of time to reach the stationary manner.

Figure 21c illustrates the influence of various $M_{1}$ values on the velocities directed in the inertial space $({{\omega_{1} } \mathord{\left/ {\vphantom {{\omega_{1} } {\omega_{3} }}} \right. \kern-0pt} {\omega_{3} }},{{\omega_{2} } \mathord{\left/ {\vphantom {{\omega_{2} } {\omega_{3} }}} \right. \kern-0pt} {\omega_{3} }})$. It is important to note that the curves depicted in Fig. 21c are characterized as directed spirals converging towards a single fixed point. This particular attribute signifies their inherent stability.

### Transverse and axial displacement solutions

In order to obtain significant insights into the body’s position in space during maneuvers near other bodies, examinations have been conducted on its transverse and axial displacement. By integrating Eq. (43) concerning time, the equation for transverse displacements can be derived, signifying

55 $$\eta (t) = \eta (0) + \int\limits_{0}^{t} {\omega (\tau )d\tau } ,$$

or

56 $$\eta (t) = \eta (0) + \int\limits_{0}^{t} {\omega_{1} (\tau )d\tau + i\int\limits_{0}^{t} {\omega_{2} (\tau )d\tau } } ,$$

where

57 $$\eta = \eta_{1} + i\eta_{2} .$$

Substituting (43) into (55), yields

58 $$\begin{aligned} \eta (t) =& \eta (0) + \int\limits_{0}^{t} {\Bigg[\omega (0) + im^{ - 1} e^{{i\,\theta_{0} }} \Bigg\{ r_{0}^{ - 1} f(1 - e^{{i\,r_{0} \tau }} ) - f_{3} \Bigg[\Gamma_{0} t + I_{\omega } (\tau )\Bigg]\Bigg\} \Bigg]d\tau } \hfill \\=& \eta (0) + \Bigg[\omega (0) + i(mr_{0} )^{ - 1} e^{{i\,\theta_{0} }} f\Bigg]t + m^{ - 1} e^{{i\,\theta_{0} }} \Bigg\{ r_{0}^{ - 2} f(e^{{i\,r_{0} t}} - 1) - if_{3} \Bigg[({{\Gamma_{0} t^{2} } \mathord{\left/ {\vphantom {{\Gamma_{0} t^{2} } {2)}}} \right. \kern-0pt} {2)}} + I_{\eta } (t)\Bigg]\Bigg\} , \hfill \\ \end{aligned}$$

where

59 $$I_{\eta } (t) = {{\Bigg[(\sqrt {\zeta_{1} } + \sqrt {\zeta_{2} } )I_{1\eta } (t) + (\sqrt {\zeta_{1} } - \sqrt {\zeta_{2} } )I_{2\eta } (t)\Bigg]} \mathord{\left/ {\vphantom {{\Bigg[(\sqrt {\zeta_{1} } + \sqrt {\zeta_{2} } )I_{1\eta } (t) + (\sqrt {\zeta_{1} } - \sqrt {\zeta_{2} } )I_{2\eta } (t)\Bigg]} {2\zeta }}} \right. \kern-0pt} {2\zeta }},$$

and

60 $$\begin{aligned} I_{1\eta } (t) =& \int\limits_{0}^{t} {I_{1\omega } (\tau )d\tau } \hfill \\ =& \frac{{ - i\Pi_{10} }}{{\zeta \Bigg[{{r_{0} + \Bigg\{ (\lambda_{3} \, - eHl^{2} \cos \sigma )} \mathord{\left/ {\vphantom {{r_{0} + \Bigg\{ (\lambda_{3} \, - eHl^{2} \cos \sigma )} {\zeta_{1} D_{1} }}} \right. \kern-0pt} {\zeta_{1} D_{1} }}\Bigg\} \Bigg] + r_{0} }}\Bigg\{ \frac{i}{{\zeta \Bigg[{{r_{0} + \Bigg\{ (\lambda_{3} \, - eHl^{2} \cos \sigma )} \mathord{\left/ {\vphantom {{r_{0} + \Bigg\{ (\lambda_{3} \, - eHl^{2} \cos \sigma )} {\zeta_{1} D_{1} }}} \right. \kern-0pt} {\zeta_{1} D_{1} }}\Bigg\} \Bigg] + r_{0} }} \hfill \\ & \times \Bigg[t + \frac{{i(e^{{it(\zeta \Bigg[{{r_{0} + \Bigg\{ (\lambda_{3} \, - eHl^{2} \cos \sigma )} \mathord{\left/ {\vphantom {{r_{0} + \Bigg\{ (\lambda_{3} \, - eHl^{2} \cos \sigma )} {\zeta_{1} D_{1} }}} \right. \kern-0pt} {\zeta_{1} D_{1} }}\Bigg\} \Bigg] + r_{0} )\,}} - 1)}}{{\zeta \Bigg[{{r_{0} + \Bigg\{ (\lambda_{3} \, - eHl^{2} \cos \sigma )} \mathord{\left/ {\vphantom {{r_{0} + \Bigg\{ (\lambda_{3} \, - eHl^{2} \cos \sigma )} {\zeta_{1} D_{1} }}} \right. \kern-0pt} {\zeta_{1} D_{1} }}\Bigg\} \Bigg] + r_{0} }}\Bigg] - \frac{{t^{2} }}{2}\Bigg\} + \frac{A}{{\zeta \Bigg[{{r_{0} + \Bigg\{ (\lambda_{3} \, - eHl^{2} \cos \sigma )} \mathord{\left/ {\vphantom {{r_{0} + \Bigg\{ (\lambda_{3} \, - eHl^{2} \cos \sigma )} {\zeta_{1} D_{1} }}} \right. \kern-0pt} {\zeta_{1} D_{1} }}\Bigg\} \Bigg]}} \hfill \\ & \times \Bigg\{ r_{0}^{ - 1} \Bigg[ir_{0}^{ - 1} \Bigg\{ t - ir_{0}^{ - 1} (1 - e^{{ir_{0} \,t}} )\Bigg\} - \frac{{t^{2} }}{2}\Bigg] - \frac{1}{{\zeta \Bigg[{{r_{0} + \Bigg\{ (\lambda_{3} \, - eHl^{2} \cos \sigma )} \mathord{\left/ {\vphantom {{r_{0} + \Bigg\{ (\lambda_{3} \, - eHl^{2} \cos \sigma )} {\zeta_{1} D_{1} }}} \right. \kern-0pt} {\zeta_{1} D_{1} }}\Bigg\} \Bigg] + r_{0} }}\Bigg[ - \frac{{t^{2} }}{2} \hfill \\ & + \frac{i}{{\zeta \Bigg[{{r_{0} + \Bigg\{ (\lambda_{3} \, - eHl^{2} \cos \sigma )} \mathord{\left/ {\vphantom {{r_{0} + \Bigg\{ (\lambda_{3} \, - eHl^{2} \cos \sigma )} {\zeta_{1} D_{1} }}} \right. \kern-0pt} {\zeta_{1} D_{1} }}\Bigg\} \Bigg] + r_{0} }}\Bigg[t + \frac{{i(e^{{it(\zeta \Bigg[{{r_{0} + \Bigg\{ (\lambda_{3} \, - eHl^{2} \cos \sigma )} \mathord{\left/ {\vphantom {{r_{0} + \Bigg\{ (\lambda_{3} \, - eHl^{2} \cos \sigma )} {\zeta_{1} D_{1} }}} \right. \kern-0pt} {\zeta_{1} D_{1} }}\Bigg\} \Bigg] + r_{0} )\,}} - 1)}}{{\zeta \Bigg[{{r_{0} + \Bigg\{ (\lambda_{3} \, - eHl^{2} \cos \sigma )} \mathord{\left/ {\vphantom {{r_{0} + \Bigg\{ (\lambda_{3} \, - eHl^{2} \cos \sigma )} {\zeta_{1} D_{1} }}} \right. \kern-0pt} {\zeta_{1} D_{1} }}\Bigg\} \Bigg] + r_{0} }}\Bigg]\Bigg\} , \hfill \\ \end{aligned}$$

61 $$\begin{aligned} I_{2\eta } (t) =& \int\limits_{0}^{t} {I_{2\omega } (\tau )d\tau } \hfill \\ =& \frac{{ - i\,\tilde{\Pi }_{10} }}{{r_{0} - \zeta \Bigg[{{r_{0} + \Bigg\{ (\lambda_{3} \, - eHl^{2} \cos \sigma )} \mathord{\left/ {\vphantom {{r_{0} + \Bigg\{ (\lambda_{3} \, - eHl^{2} \cos \sigma )} {\zeta_{1} D_{1} \Bigg\} }}} \right. \kern-0pt} {\zeta_{1} D_{1} \Bigg\} }}\Bigg]}}\Bigg\{ \frac{i}{{r_{0} - \zeta \Bigg[{{r_{0} + \Bigg\{ (\lambda_{3} \, - eHl^{2} \cos \sigma )} \mathord{\left/ {\vphantom {{r_{0} + \Bigg\{ (\lambda_{3} \, - eHl^{2} \cos \sigma )} {\zeta_{1} D_{1} \Bigg\} }}} \right. \kern-0pt} {\zeta_{1} D_{1} \Bigg\} }}\Bigg]}} \hfill \\ & \times \Bigg[t + \frac{{i(1 - e^{{it(r_{0} - \zeta \Bigg[{{r_{0} + \Bigg\{ (\lambda_{3} \, - eHl^{2} \cos \sigma )} \mathord{\left/ {\vphantom {{r_{0} + \Bigg\{ (\lambda_{3} \, - eHl^{2} \cos \sigma )} {\zeta_{1} D_{1} }}} \right. \kern-0pt} {\zeta_{1} D_{1} }}\Bigg\} \Bigg])}} - 1)}}{{r_{0} - \zeta \Bigg[{{r_{0} + \Bigg\{ (\lambda_{3} \, - eHl^{2} \cos \sigma )} \mathord{\left/ {\vphantom {{r_{0} + \Bigg\{ (\lambda_{3} \, - eHl^{2} \cos \sigma )} {\zeta_{1} D_{1} \Bigg\} }}} \right. \kern-0pt} {\zeta_{1} D_{1} \Bigg\} }}\Bigg]}}\Bigg] - \frac{{t^{2} }}{2}\Bigg\} - \frac{{\tilde{A}}}{{\zeta \Bigg[{{r_{0} + \Bigg\{ (\lambda_{3} \, - eHl^{2} \cos \sigma )} \mathord{\left/ {\vphantom {{r_{0} + \Bigg\{ (\lambda_{3} \, - eHl^{2} \cos \sigma )} {\zeta_{1} D_{1} \Bigg\} }}} \right. \kern-0pt} {\zeta_{1} D_{1} \Bigg\} }}\Bigg]}} \hfill \\ & \times \Bigg\{ r_{0}^{ - 1} \Bigg[ir_{0}^{ - 1} \Bigg\{ t - ir_{0}^{ - 1} (1 - e^{{ir_{0} \,t}} )\Bigg\} - \frac{{t^{2} }}{2}\Bigg] - \frac{1}{{r_{0} - \zeta \Bigg[{{r_{0} + \Bigg\{ (\lambda_{3} \, - eHl^{2} \cos \sigma )} \mathord{\left/ {\vphantom {{r_{0} + \Bigg\{ (\lambda_{3} \, - eHl^{2} \cos \sigma )} {\zeta_{1} D_{1} \Bigg\} }}} \right. \kern-0pt} {\zeta_{1} D_{1} \Bigg\} }}\Bigg]}}\Bigg[ - \frac{{t^{2} }}{2} \hfill \\ & + \frac{i}{{r_{0} - \zeta \Bigg[{{r_{0} + \Bigg\{ (\lambda_{3} \, - eHl^{2} \cos \sigma )} \mathord{\left/ {\vphantom {{r_{0} + \Bigg\{ (\lambda_{3} \, - eHl^{2} \cos \sigma )} {\zeta_{1} D_{1} \Bigg\} }}} \right. \kern-0pt} {\zeta_{1} D_{1} \Bigg\} }}\Bigg]}}\Bigg[t + \frac{{i(1 - e^{{it(r_{0} - \zeta \Bigg[{{r_{0} + \Bigg\{ (\lambda_{3} \, - eHl^{2} \cos \sigma )} \mathord{\left/ {\vphantom {{r_{0} + \Bigg\{ (\lambda_{3} \, - eHl^{2} \cos \sigma )} {\zeta_{1} D_{1} }}} \right. \kern-0pt} {\zeta_{1} D_{1} }}\Bigg\} \Bigg])}} - 1)}}{{r_{0} - \zeta \Bigg[{{r_{0} + \Bigg\{ (\lambda_{3} \, - eHl^{2} \cos \sigma )} \mathord{\left/ {\vphantom {{r_{0} + \Bigg\{ (\lambda_{3} \, - eHl^{2} \cos \sigma )} {\zeta_{1} D_{1} \Bigg\} }}} \right. \kern-0pt} {\zeta_{1} D_{1} \Bigg\} }}\Bigg]}}\Bigg]\Bigg\} . \hfill \\ \end{aligned}$$

In a manner akin to the scenario involving transverse displacements, the axial displacement can be determined by integrating Eq. (47) to get

62 $$\eta_{3} (t) = \eta_{3} (0) + \int\limits_{0}^{t} {\omega_{3} (\tau )d\tau } ,$$

or

63 $$\eta_{3} (t) = \eta_{3} (0) + \omega_{3} (0)t + \frac{{f_{3} }}{2m}t^{2} + \frac{i}{2m}(\tilde{f}\,I_{3\eta } - f\,\tilde{I}_{3\eta } ),$$

where

64 $$\begin{aligned} I_{3\eta } (t) = & \int\limits_{0}^{t} {\Bigg[ { - \frac{{i\,\Gamma_{0} }}{{r_{0} }}(1 - e^{{ - ir_{0} \,\tau }} ) + \,I_{3\omega } (t)} \Bigg]d\tau } \\ = & \frac{{i\Gamma_{0} }}{{r_{0} }}\Bigg[ {\frac{{i(e^{{ - ir_{0} \,t}} - 1)}}{{r_{0} }} - t} \Bigg] + \zeta_{3} I_{4\eta } (t) + \zeta_{4} I_{5\eta } (t), \\ \end{aligned}$$

65 $$\begin{aligned} I_{4\eta } (t) =& \frac{{ - i\Pi_{10} }}{{\zeta \Bigg[{{r_{0} + \Bigg\{ (\lambda_{3} \, - eHl^{2} \cos \sigma )} \mathord{\left/ {\vphantom {{r_{0} + \Bigg\{ (\lambda_{3} \, - eHl^{2} \cos \sigma )} {\zeta_{1} D_{1} }}} \right. \kern-0pt} {\zeta_{1} D_{1} }}\Bigg\} \Bigg] + r_{0} }}\Bigg\{ \frac{i}{{\zeta \Bigg[{{r_{0} + \Bigg\{ (\lambda_{3} \, - eHl^{2} \cos \sigma )} \mathord{\left/ {\vphantom {{r_{0} + \Bigg\{ (\lambda_{3} \, - eHl^{2} \cos \sigma )} {\zeta_{1} D_{1} }}} \right. \kern-0pt} {\zeta_{1} D_{1} }}\Bigg\} \Bigg] + r_{0} }} \hfill \\ & \times \Bigg[t + \frac{{i(e^{{it\Bigg\{ {{\zeta \Bigg[r_{0} + \Bigg\{ (\lambda_{3} \, - eHl^{2} \cos \sigma )} \mathord{\left/ {\vphantom {{\zeta \Bigg[r_{0} + \Bigg\{ (\lambda_{3} \, - eHl^{2} \cos \sigma )} {\zeta_{1} D_{1} }}} \right. \kern-0pt} {\zeta_{1} D_{1} }}\Bigg\} \Bigg] + r_{0} \Bigg\} \,}} - 1\Bigg]}}{{\zeta \Bigg[{{r_{0} + \Bigg\{ (\lambda_{3} \, - eHl^{2} \cos \sigma )} \mathord{\left/ {\vphantom {{r_{0} + \Bigg\{ (\lambda_{3} \, - eHl^{2} \cos \sigma )} {\zeta_{1} D_{1} }}} \right. \kern-0pt} {\zeta_{1} D_{1} }}\Bigg\} \Bigg] + r_{0} }}\Bigg] - \frac{{t^{2} }}{2}\Bigg\} + \frac{A}{{\zeta \Bigg[{{r_{0} + \Bigg\{ (\lambda_{3} \, - eHl^{2} \cos \sigma )} \mathord{\left/ {\vphantom {{r_{0} + \Bigg\{ (\lambda_{3} \, - eHl^{2} \cos \sigma )} {\zeta_{1} D_{1} }}} \right. \kern-0pt} {\zeta_{1} D_{1} }}\Bigg\} \Bigg]}} \hfill \\ & \times \Bigg\{ r_{0}^{ - 1} \Bigg[ir_{0}^{ - 1} \Bigg\{ t - ir_{0}^{ - 1} (1 - e^{{ir_{0} \,t}} )\Bigg\} - \frac{{t^{2} }}{2}\Bigg] - \frac{1}{{\zeta \Bigg[{{r_{0} + \Bigg\{ (\lambda_{3} \, - eHl^{2} \cos \sigma )} \mathord{\left/ {\vphantom {{r_{0} + \Bigg\{ (\lambda_{3} \, - eHl^{2} \cos \sigma )} {\zeta_{1} D_{1} }}} \right. \kern-0pt} {\zeta_{1} D_{1} }}\Bigg\} \Bigg] + r_{0} }}\Bigg[ - \frac{{t^{2} }}{2} \hfill \\ & + \frac{i}{{\zeta \Bigg[{{r_{0} + \Bigg\{ (\lambda_{3} \, - eHl^{2} \cos \sigma )} \mathord{\left/ {\vphantom {{r_{0} + \Bigg\{ (\lambda_{3} \, - eHl^{2} \cos \sigma )} {\zeta_{1} D_{1} }}} \right. \kern-0pt} {\zeta_{1} D_{1} }}\Bigg\} \Bigg] + r_{0} }}\Bigg[t + \frac{{i(e^{{it\Bigg\{ {{\zeta \Bigg[r_{0} + \Bigg\{ (\lambda_{3} \, - eHl^{2} \cos \sigma )} \mathord{\left/ {\vphantom {{\zeta \Bigg[r_{0} + \Bigg\{ (\lambda_{3} \, - eHl^{2} \cos \sigma )} {\zeta_{1} D_{1} }}} \right. \kern-0pt} {\zeta_{1} D_{1} }}\Bigg\} \Bigg] + r_{0} \Bigg\} \,}} - 1)}}{{\zeta \Bigg[{{r_{0} + \Bigg\{ (\lambda_{3} \, - eHl^{2} \cos \sigma )} \mathord{\left/ {\vphantom {{r_{0} + \Bigg\{ (\lambda_{3} \, - eHl^{2} \cos \sigma )} {\zeta_{1} D_{1} }}} \right. \kern-0pt} {\zeta_{1} D_{1} }}\Bigg\} \Bigg] + r_{0} }}\Bigg]\Bigg\} , \hfill \\ \end{aligned}$$

66 $$\begin{aligned} I_{5\eta } (t) =& \frac{{ - i\Pi_{10} }}{{r_{0} - \zeta \Bigg[{{r_{0} + \Bigg\{ (\lambda_{3} \, - eHl^{2} \cos \sigma )} \mathord{\left/ {\vphantom {{r_{0} + \Bigg\{ (\lambda_{3} \, - eHl^{2} \cos \sigma )} {\zeta_{1} D_{1} }}} \right. \kern-0pt} {\zeta_{1} D_{1} }}\Bigg\} \Bigg]}}\Bigg\{ \frac{i}{{r_{0} - \zeta \Bigg[{{r_{0} + \Bigg\{ (\lambda_{3} \, - eHl^{2} \cos \sigma )} \mathord{\left/ {\vphantom {{r_{0} + \Bigg\{ (\lambda_{3} \, - eHl^{2} \cos \sigma )} {\zeta_{1} D_{1} }}} \right. \kern-0pt} {\zeta_{1} D_{1} }}\Bigg\} \Bigg]}} \hfill \\ & \times \Bigg[t + \frac{{i(1 - e^{{it\Bigg\{ {{r_{0} - \zeta \Bigg[r_{0} + \Bigg\{ (\lambda_{3} \, - eHl^{2} \cos \sigma )} \mathord{\left/ {\vphantom {{r_{0} - \zeta \Bigg[r_{0} + \Bigg\{ (\lambda_{3} \, - eHl^{2} \cos \sigma )} {\zeta_{1} D_{1} \Bigg\} }}} \right. \kern-0pt} {\zeta_{1} D_{1} \Bigg\} }}\Bigg]\Bigg\} \,}} \Bigg]}}{{r_{0} - \zeta \Bigg[{{r_{0} + \Bigg\{ (\lambda_{3} \, - eHl^{2} \cos \sigma )} \mathord{\left/ {\vphantom {{r_{0} + \Bigg\{ (\lambda_{3} \, - eHl^{2} \cos \sigma )} {\zeta_{1} D_{1} }}} \right. \kern-0pt} {\zeta_{1} D_{1} }}\Bigg\} \Bigg]}}\Bigg] - \frac{{t^{2} }}{2}\Bigg\} - \frac{{\tilde{A}}}{{\zeta \Bigg[{{r_{0} + \Bigg\{ (\lambda_{3} \, - eHl^{2} \cos \sigma )} \mathord{\left/ {\vphantom {{r_{0} + \Bigg\{ (\lambda_{3} \, - eHl^{2} \cos \sigma )} {\zeta_{1} D_{1} }}} \right. \kern-0pt} {\zeta_{1} D_{1} }}\Bigg\} \Bigg]}} \hfill \\ & \times \Bigg\{ r_{0}^{ - 1} \Bigg[ir_{0}^{ - 1} \Bigg\{ t - ir_{0}^{ - 1} (1 - e^{{ir_{0} \,t}} )\Bigg\} - \frac{{t^{2} }}{2}\Bigg] - \frac{1}{{r_{0} - \zeta \Bigg[{{r_{0} + \Bigg\{ (\lambda_{3} \, - eHl^{2} \cos \sigma )} \mathord{\left/ {\vphantom {{r_{0} + \Bigg\{ (\lambda_{3} \, - eHl^{2} \cos \sigma )} {\zeta_{1} D_{1} }}} \right. \kern-0pt} {\zeta_{1} D_{1} }}\Bigg\} \Bigg]}}\Bigg[ - \frac{{t^{2} }}{2} \hfill \\ & + \frac{i}{{r_{0} - \zeta \Bigg[{{r_{0} + \Bigg\{ (\lambda_{3} \, - eHl^{2} \cos \sigma )} \mathord{\left/ {\vphantom {{r_{0} + \Bigg\{ (\lambda_{3} \, - eHl^{2} \cos \sigma )} {\zeta_{1} D_{1} }}} \right. \kern-0pt} {\zeta_{1} D_{1} }}\Bigg\} \Bigg]}}(t + \frac{{i(1 - e^{{it\Bigg\{ {{r_{0} - \zeta \Bigg[r_{0} + \Bigg\{ (\lambda_{3} \, - eHl^{2} \cos \sigma )} \mathord{\left/ {\vphantom {{r_{0} - \zeta \Bigg[r_{0} + \Bigg\{ (\lambda_{3} \, - eHl^{2} \cos \sigma )} {\zeta_{1} D_{1} \Bigg\} }}} \right. \kern-0pt} {\zeta_{1} D_{1} \Bigg\} }}\Bigg]\Bigg\} \,}} )}}{{r_{0} - \zeta \Bigg[{{r_{0} + \Bigg\{ (\lambda_{3} \, - eHl^{2} \cos \sigma )} \mathord{\left/ {\vphantom {{r_{0} + \Bigg\{ (\lambda_{3} \, - eHl^{2} \cos \sigma )} {\zeta_{1} D_{1} }}} \right. \kern-0pt} {\zeta_{1} D_{1} }}\Bigg\} \Bigg]}})\Bigg\} , \hfill \\ \end{aligned}$$

whereas $\tilde{I}_{3\eta }$ is the complex conjugate of the function $I_{3\eta }$.

As anticipated, the axial displacement exhibits secular terms, as Eqs. (65) and (66) are derived through the integration of Eqs. (50) and (51), respectively. These secular terms correspond to both linear and parabolic characteristics.

**Figure 22:**
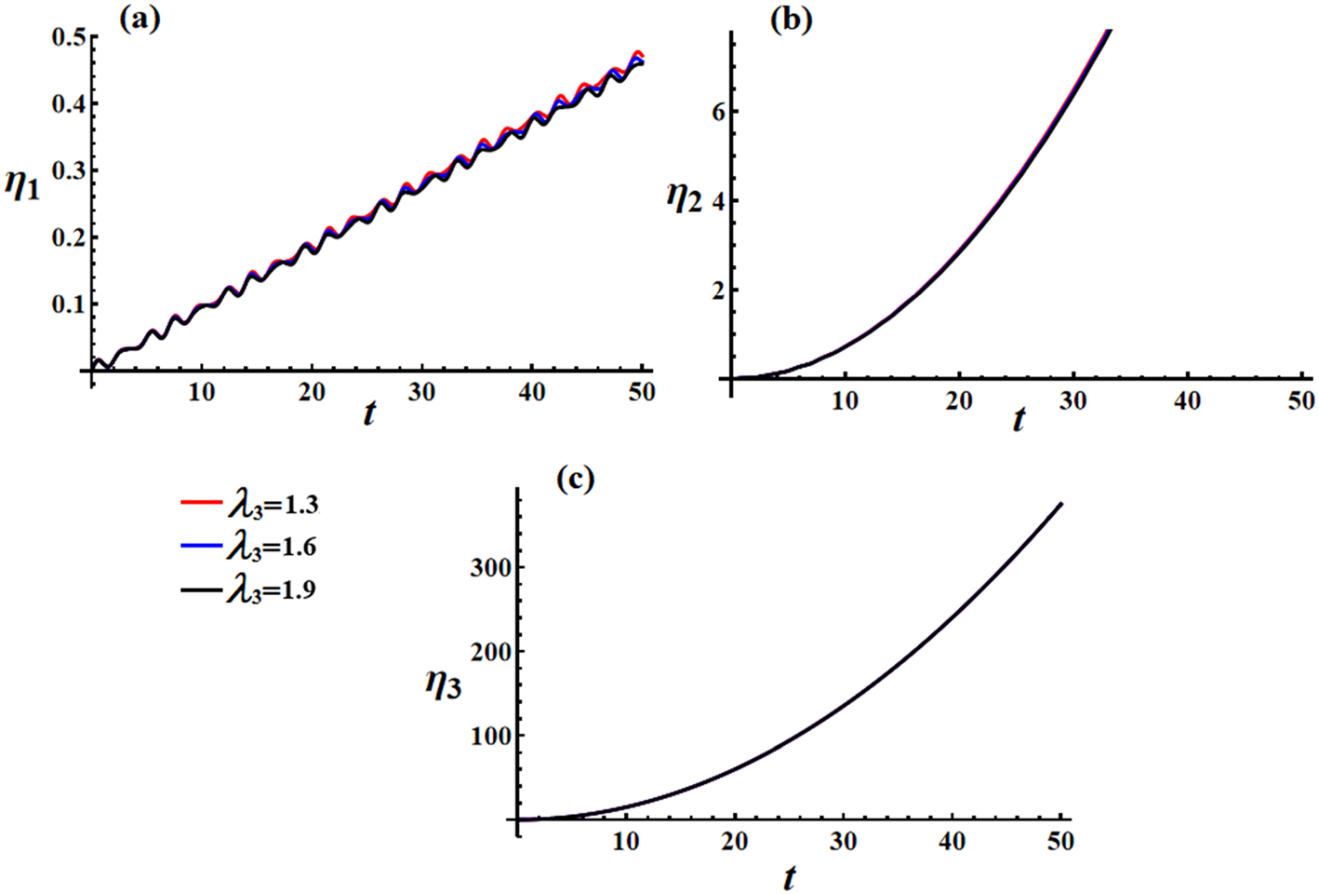
Represents AS for $\eta_{1}$, $\eta_{2}$ and $\eta_{3}$ at $e = 0.003\,{\text{C,}}\,\,M_{1} = 4.0\,{\text{N}}\,{\text{m}}$ when $\lambda_{3} ( = 1.3,1.6,1.9)\,{\text{kg}}\,{\text{m}}^{2} \,{\text{s}}^{ - 1}$.

Figure 22 displays the AS for transverse displacements $\eta_{1}$ and $\eta_{2}$, as well as the axial displacement $\eta_{3}$. These solutions are obtained by using the real and imaginary parts of Eq. (48) for the transverse displacements, whereas Eq. (62) represents the axial displacement. The alteration in the GM leads to a variation in the value of $\eta_{1}$, which is owing to the slight decrease in linear growth of the $\eta_{1}$ waves. On the other hand, the behavior of $\eta_{2}$ decreases slightly over time, eventually is converging towards the same parabolic growth as its values. The GM, however, does not have any effect on the parabolic growth of $\eta_{3}$ which is due to the absence of the torques in the solution for $\eta_{3}$ in Eq. (63) which has been approved through the simulation.

**Figure 23:**
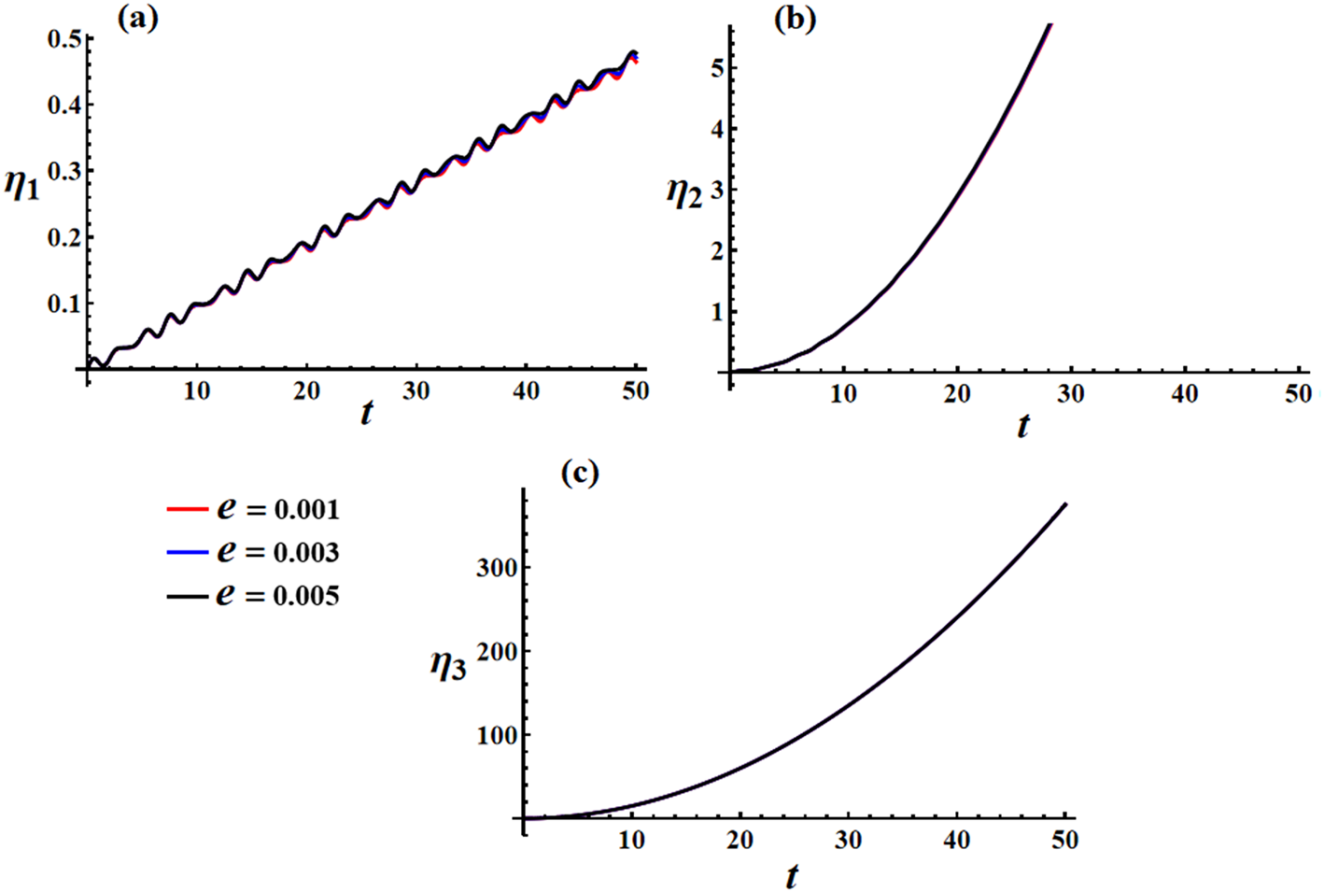
Demonstrates the obtained solution for $\eta_{1}$, $\eta_{2}$ and $\eta_{3}$ at $\lambda_{3} = 1.3\,{\text{kg}}\,{\text{m}}^{2} \,{\text{s}}^{ - 1} ,\,M_{1} = 4.0\,{\text{N}}\,{\text{m}}$ when $e( = 0.001,0.003,0.005){\text{C}}$.

The time behavior of $\eta_{1} ,\eta_{2} ,$ and $\eta_{3}$ is shown in Fig. 23 at $\lambda_{3} = 1.3\,{\text{kg}}\,{\text{m}}^{2} \,{\text{s}}^{ - 1} ,\,M_{1} = 4.0\,{\text{N}}\,{\text{m}}$ when $e( = 0.001,0.003,0.005){\text{C}}$ in addition to the other aforementioned data. It is clear that the behavior of $\eta_{1}$ increases gradually with an observed oscillation of the represented waves when $e$ varies, as seen in Fig. 23a. A slight growth rate of $\eta_{2}$ waves without oscillations is observed in Fig. 23b to reach the maximum value during a shorter time interval. However, the charge does not have any impact on the parabolic growth of $\eta_{3}$, as demonstrated in Fig. 23c.

**Figure 24:**
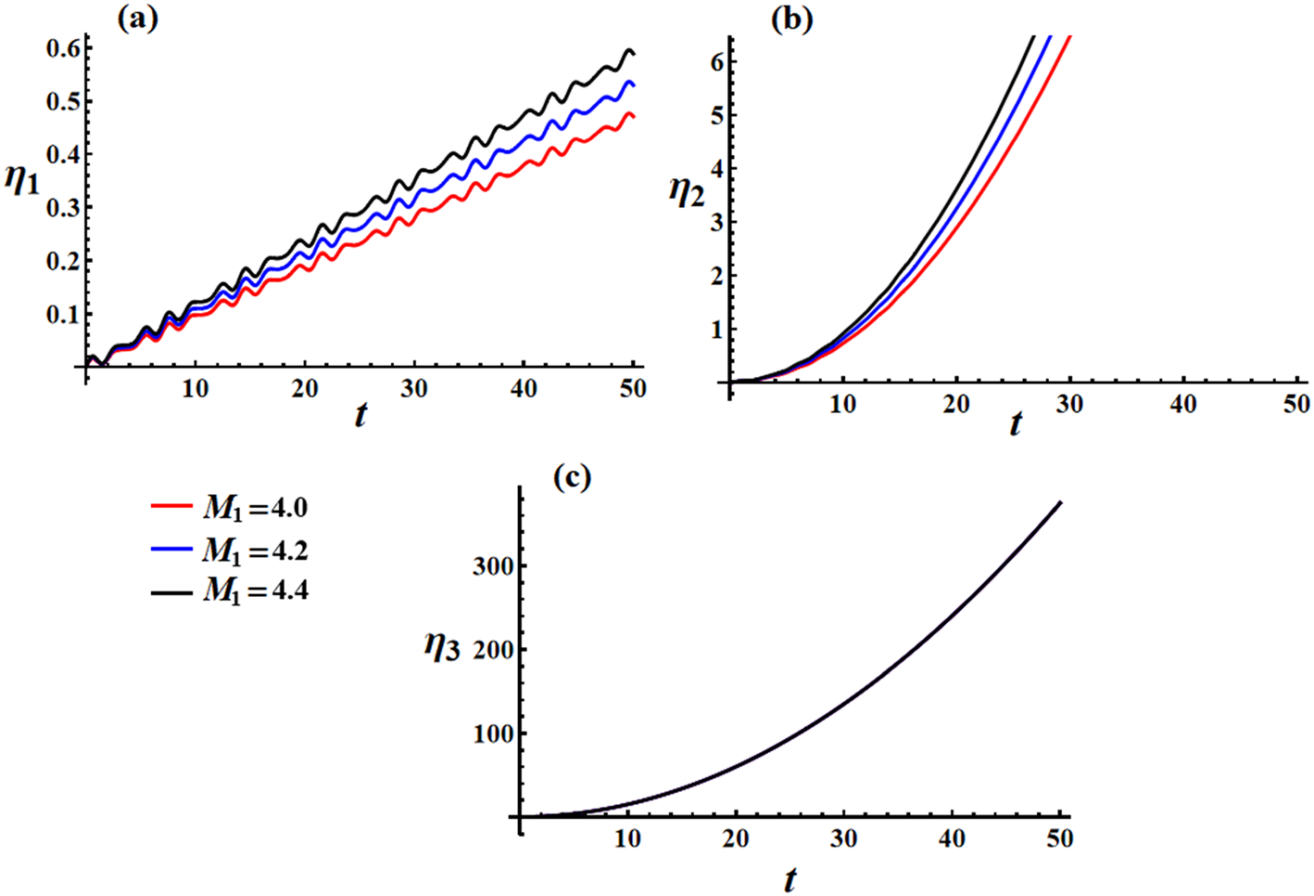
Showcases the achieved solution for $\eta_{1} ,\eta_{2} ,$ and $\eta_{3}$ at $\lambda_{3} = 1.3\,{\text{kg}}\,{\text{m}}^{2} \,{\text{s}}^{ - 1} {,}\,\,e = 0.003{\text{C}}$ when $M_{1} ( = 4.0,4.5,5.0)\,{\text{N}}\,{\text{m}}$.

The curves in Fig. 24 illustrate the influence of $M_{1}$ on the solutions $\eta_{1} ,\eta_{2} ,$ and $\eta_{3}$. When $M_{1}$ is increased, the waves representing $\eta_{1}$ also increase throughout the time interval. This is due to the linear dependency of $\eta_{1}$ on time. On the other hand, $\eta_{2}$ experiences a gradual increase and achieves a predetermined value during the final quarter of this interval. As expected, the changes in $M_{1}$ do not impact the parabolic growth of $\eta_{3}$.

Since^4^ closely aligned with our research topic and yielded identical results with zero error between techniques, we set both the GM and charge to zero for comparison. This was done to validate the accuracy of our investigative methodology, particularly in light of the lack of previous studies examining the effects of GM and charge. By referencing these similar studies, we sought to affirm the correctness of our approach.

## Conclusion

The present study has been focused on achieving the novel analytic solution for the motion of a spinning axisymmetric charged RB. Therefore, Euler’s equations have been utilized to derive the EOM that governs the RB’s rotatory motion. Due to the absence of torque along the spin axis and the RB possesses an almost axisymmetric characteristic, the spin rate remains relatively stable. The AS of the RB for the attitude, rotational, and translational motion have been derived through an assumption of small angular deviations of the spin axis with respect to a fixed direction in space. These solutions encompass various parameters such as angular velocities, Euler’s angles, angular momentum, transverse velocities, transverse displacements, axial velocity, and axial displacement. The influence of the GM, electromagnetic force field, and a fixed torque, which is constant concerning the RB’s frame, on its motion, have been examined. For the studied case of the GM, increasing the value of the GM implies a decrease in the angular velocities’ components, precession and proper rotation angles, angular momentum, transverse and axial velocities, and transverse and axial displacements. On the other hand, the increase in both the charge and the constant torque implies an increase in all the aforementioned parameters. These phenomena could be used to maintain the motion of the RB after the increasing or the decreasing of the external torque applied to it for such the body remain confined to a known region which remain stable on it. The combination of these effects gives a similar effect to each effect separately. These results are the outcome for the specific condition used to study these effects. To validate the accuracy of the employed method, graphical simulations of these solutions have been presented. Additionally, a computer program is utilized to visualize the results and demonstrate how different values for the RB’s parameters affect the behavior of its motion. The resulting diagrams include phase plane curves that illustrate the influence of the GM, electromagnetic force field, and IBFT on the RB’s motion. The phase plane diagrams also serve as a means to give an induction about the stability of the motion for specific cases where initial conditions are applied. This research is of great significance in numerous scientific and engineering fields as it can optimize mechanical systems, elucidate celestial motion, and improve the performance of machines in the space industrial field.

## Data Availability

All data generated or analysed during this study are included in this published article.
